# Rab26 suppresses migration and invasion of breast cancer cells through mediating autophagic degradation of phosphorylated Src

**DOI:** 10.1038/s41419-021-03561-7

**Published:** 2021-03-17

**Authors:** Huiying Liu, Yuxia Zhou, Hantian Qiu, Ruijuan Zhuang, Yang Han, Xiaoqing Liu, Xi Qiu, Ziyan Wang, Liju Xu, Ran Tan, Wanjin Hong, Tuanlao Wang

**Affiliations:** 1 School of Pharmaceutical Sciences, State Key Laboratory of Cellular Stress Biology, Fujian Provincial Key Laboratory of Innovative Drug Target Research, Xiamen University, 361005 Fujian, China; 2 School of Basic Medical Sciences, Guizhou Provincial Key Laboratory of Pathogenesis and Drug Research on Common Chronic Diseases, Guizhou Medical University, 550025 Guiyang, China; 3 Institute of Molecular and Cell Biology, A STAR (Agency of ScienceTechnology and Research), 61 Biopolis Drive, Singapore, 138673 Singapore

**Keywords:** Cell migration, Small GTPases

## Abstract

Rab proteins play crucial roles in membrane trafficking. Some Rab proteins are implicated in cancer development through regulating protein sorting or degradation. In this study, we found that the expression of Rab26 is suppressed in the aggressive breast cancer cells as compared to the levels in non-invasive breast cancer cells. Over-expression of Rab26 inhibits cell migration and invasion, while Rab26 knockdown significantly promotes the migration and invasion of breast cancer cells. Rab26 reduces focal adhesion association of Src kinase and induces endosomal translocation of Src. Further experiments revealed that Rab26 mediates the autophagic degradation of phosphorylated Src through interacting with ATG16L1, consequently, resulting in the suppression of the migration and invasion ability of breast cancer cells.

## Introduction

Cell adhesion, migration, and invasion are closely associated with the cancer development which are mediated by multiple types of proteins, including adhesion proteins, surface receptors, and their scaffolds. Intrinsically, integrin, FAK, and Src kinases form adhesion complex at the plasma membrane to transit mechanic force to cytoskeleton to regulate migration of cancer cell^1–3^. Intracellular membrane trafficking events, including endocytosis, exocytosis, and autophagy, control protein sorting to correct compartments or regulate protein turnover, playing a fundamental role in maintaining membrane homeostasis and sustaining cellular signaling^4,5^. Membrane trafficking disorder links to human diseases such as cancer^6–9^. Typically, the aberrant internalization and recycling of integrin mediate the formation of focal adhesion to regulate migration and invasion of cancer cells^10,11^, and the lysosomal degradation of surface protein such as E-cadherin enhances epithelial–mesenchymal transition (EMT)^12^.

Rab small GTPases are the master regulators for membrane trafficking, serving as the molecular switches to mediate vesicle budding, translocation, docking, and fusion events^13^. About 70 Rab proteins are characterized and engaged in diverse trafficking pathways in human cells^14^. Dysregulated Rab proteins are implicated in multiple types of cancers by influencing the adhesion, motility, and invasion of cancer cells through regulating receptor endocytosis, recycling, or degradation^15,16^. Rab25 is the first identified Rab protein that is directly involved in the progress of breast cancer and ovary cancer through mediating integrin αvβ1 trafficking and regulating AKT signaling pathway^17^. Rab5 plays a key role in the early stage of endocytosis, regulating the trafficking and signal transduction of multiple membrane receptors such as EGFR, c-Met, and GPCR, thus affecting cell proliferation, apoptosis, and tumorigenesis^18^. Tyrosine phosphorylation of Rab34 by Src kinase mediates integrin internalization and promotes adhesion and migration of breast cancer cells^19^. Abnormal expressions of Rab2, Rab3, Rab13, Rab17, Rab31, and Rab35 are closely related to diverse cancers^20–25^, implicating these Rab proteins may serve as biomarkers in different cancers.

Rab26 is the transcriptional target of MIST1, involved in regulating exocrine granule maturation and amylase release from parotid acinar cells^26,27^. Several investigations uncovered multiple functions of Rab26. Rab26 induces lysosomes to aggregate at the perinuclear region and in turn causes mitochondrial redistribution^28^. Rab26 directs the autophagy pathway of synaptic vesicles by interacting with ATG16L1 (ref. ^29^). Recent studies found that Rab26 enhances the integrity of adheren junctions in acute lung injury by regulating the degradation of phosphorylated Src^30^. Rab26 also modulates the trafficking of cell surface receptor such as α2-adrenergic receptor (α2-AR) and mediates the balance between β2-AR and TLR4 (refs. ^31,32^). SNRPB promotes the tumorigenic potential of NSCLC by regulating Rab26 expression, not relying on Rab26’s autophagic regulatory function^33^, suggesting a potential role of Rab26 in tumorigenesis. Nevertheless, the role of Rab26 in cancer deserves further examinations.

In this study, we found that Rab26 is expressed at higher level in lower-invasive breast cancer cell lines, and its expression is suppressed in invasive breast cancer cells. Over-expression of Rab26 inhibits migration and invasion of invasive breast cancer cells. Further investigations revealed that Rab26 regulates the degradation of the active phospho-Src kinase through autophagic pathway, thus inhibiting cell adhesion, migration, and invasion of breast cancer cells.

## Results

### Rab26 level is suppressed in aggressive breast cancer cells

**Fig. 1:**
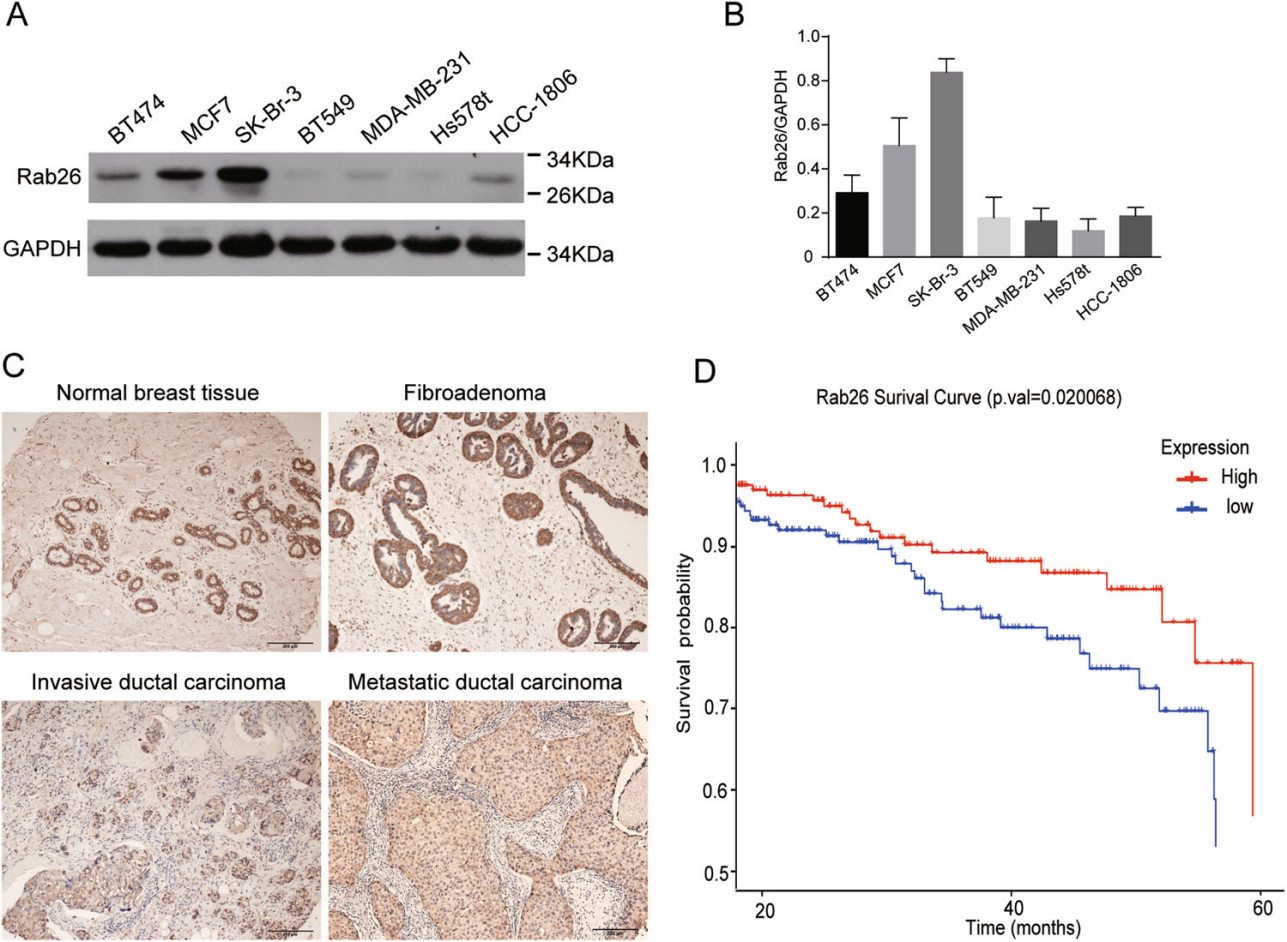
The expression of Rab26 in breast cancer. **A** Western-blot experiment demonstrated the protein levels of Rab26 in different breast cancer cell lines. **B** Quantitative analysis of from three independent experiments described in **A**, the protein level was normalized to the amount of GAPDH, the results showed Rab26 is highly expressed in non-invasive breast cancer cell lines but suppressed in the invasive breast cancer cell lines. **C** The representative IHC pictures showed the expression of Rab26 in normal breast tissues, fibroadenoma, invasive ductal carcinoma, and metastatic invasive ductal carcinoma. Bar = 200 μm. **D** The clinical significance of Rab26 expression in overall survivals was evaluated by Kaplan–Meier survival analyses (*n* = 985) based on TGCA database, *p* = 0.020068.

Through systematically examination of the mRNA levels of Rab proteins in breast cancer cell lines (data not shown), we found the expression level of Rab26 transcript is relatively lower in all eight breast cancer cell lines compared with the normal breast tissue cell line MCF10A (Fig. S1A). To further clarify the expression of Rab26 in breast cancer cells, we performed western-blot assay to examine the protein levels in different cell lines, and found that the protein levels of Rab26 are much higher in low invasive breast cancer cells MCF7, SK-Br-3, and BT474 than those in highly invasive cell lines MDA-MB-231, BT549, Hs578T, and HCC-1806 (Fig. 1A, B).

IHC analysis revealed that both normal tissues and cancer tissues have Rab26 staining signals, especially, Rab26 is preferentially expressed in the ductal carcinoma, and comparatively lower staining signal was observed in the metastatic invasive ductal cancer (Fig. 1C and Table S1). Survival analysis using breast cancer datasets showed that breast cancer patients with higher Rab26 expression are correlated with a significantly higher probability of overall survival (Fig. 1D). In consistent with the expression pattern in the breast cancer lines, these results suggested that Rab26 may play an important role in suppressing invasive behavior of breast cancer.

### Rab26 inhibits migration and invasion of breast cancer cells

**Fig. 2:**
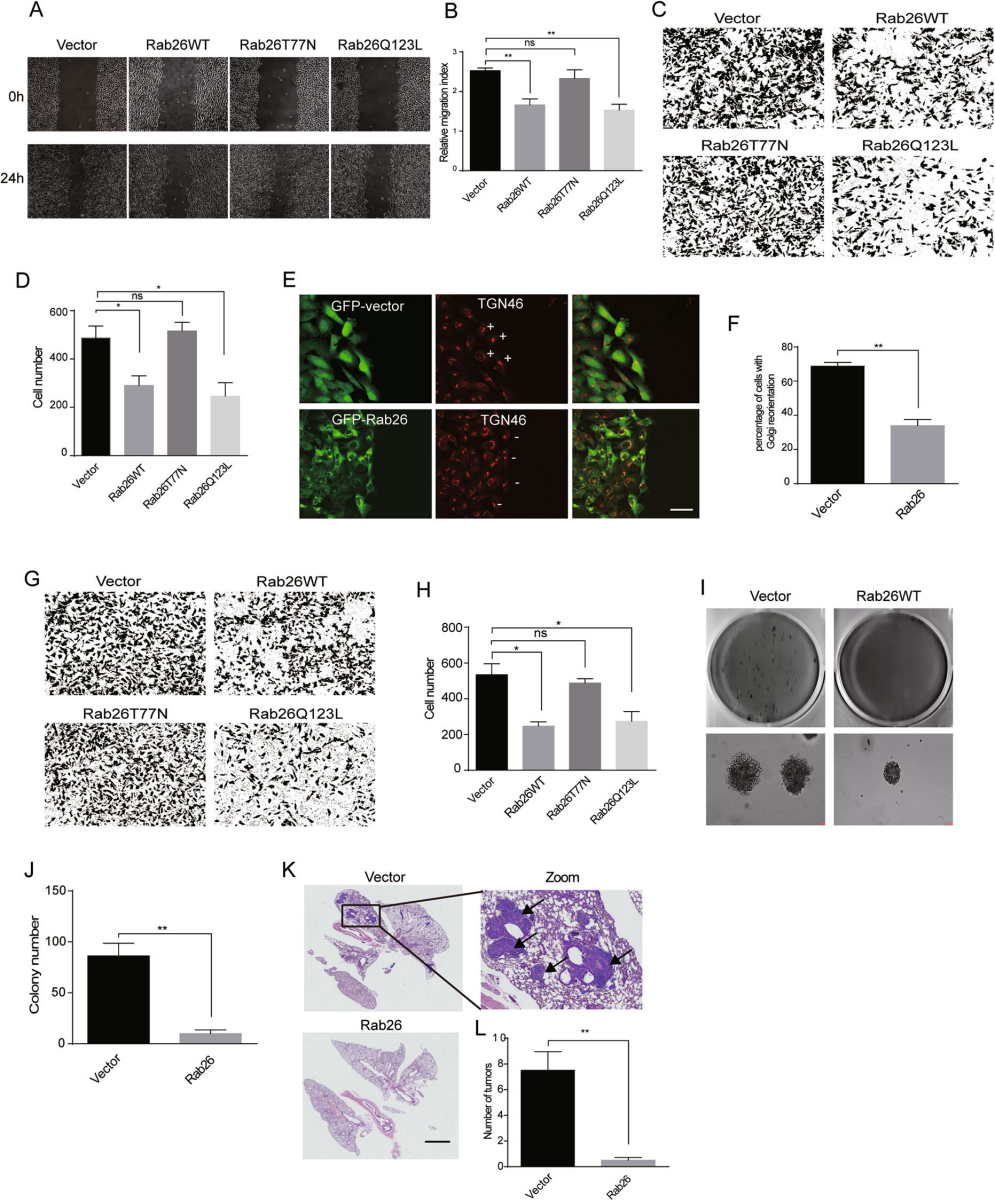
Rab26 Inhibits migration and invasion of breast cancer cells. **A** Wound-healing assay revealed that over-expression of Rab26 inhibits migration of MDA-MB-231 cells. **B** Quantitative analysis of the wound area using imageJ software from three independent wound-healing experiments, **p < 0.01. **C** Transwell assays showed Rab26 over-expression inhibits migration of MDA-MB-231 cells. **D** Quantitative analysis of the results of **C** from three independent experiments, *p < 0.05. **E** Rab26 alters Golgi reorientation during migration in MDA-MB-231 cells. The Golgi apparatus was labeled with TGN46 antibody, bar = 20 μm. “+” and “−” means the Golgi facing in the direction of migration or not, respectively. Bar = 50 μm. **F** Quantitative analysis of the results of **E** from three independent experiments, **p < 0.01. **G** Matrigel assay showed Rab26 inhibits invasive ability of MDA-MB-231 cells. **H** Quantitative analysis of the results of **G** from three independent experiments, *p < 0.05. **I** Over-expression of Rab26 inhibits colony formation of MDA-MB-231 cells in soft agar. **J** Quantitative analysis of **I** from three independent experiments, **p < 0.01. **K** HE staining demonstrated that over-expression of Rab26 inhibits the metastasis of MDA-MB-231 cells and tumor formation in lung tissues of nude mice. Arrows indicates the tumors, bar = 3000 μm. **L** Quantitative analysis of **K**, *n* = 5. **p < 0.01.

Since Rab26 is lower expressed in the high-aggressive breast cancer cells, we examined the possibility that over-expression of Rab26 will inhibit the migration and invasion of the highly invasive breast cancer cells. MDA-MB-231 cells stably expressing Rab26 or vector were generated through lentivirus-mediated expression system (Fig. S1B). Wound-healing assays revealed that over-expression of Rab26 obviously suppressed the migration of MDA-MB-231 cells (Fig. 2A, B), which was confirmed by transwell migration assays (Fig. 2C, D). Importantly, this inhibition is functionally in dependent of the guanine nucleotide-binding activity of Rab26, in that the dominant-negative mutant Rab26T77N (preferring binding to GDP) did not inhibit the cell migration, while the constitutive active mutant Rab26Q123L (lacking GTPase activity) still inhibited the cell migration (Fig. 2A, C).

In the cell wound-healing experiments, Golgi apparatus marked with TGN46 always orientates at the leading side of the migrating control MDA-MB-231 cells at the wound edge (Fig. 2E, upper panels); however, over-expression of Rab26 significantly reduces this orientation of Golgi and resulted in random orientation of the Golgi apparatus to the wound edge in MDA-MB-231 cells (Fig. 2E, lower panels and Fig. 2F), indicating the inhibition of migration ability^34,35^.

Matrigel transwell assay showed that over-expression of Rab26WT or Rab26Q123L, but not Rab26T77N, significantly reduced the invading cell number (Fig. 2G, H), indicating that Rab26 inhibits the invasive ability of MDA-MB-231 cells, and this inhibition is functionally regulated by Rab26’s nucleotide-binding activity. Further experiments demonstrated that Rab26 decreases the levels of MMP2/MMP9 (Fig. S1C), which is essential to cell invasion. In addition, soft-agar assay demonstrated that over-expression of Rab26 significantly reduced the colonies of MDA-MB-231 cells on soft agar (Fig. 2I, J). To investigate the effect of Rab26 on cell migration/invasion in vivo, nude mice were tail-vein injected with MDA-MB-231 cells expressing Rab26 or vector, and tumor formation in lung tissue was examined by hematoxylin (HE) staining. The results revealed that control cells result in severe tumor formation in the lung; however, Rab26 significantly reduced the tumor number (Fig. 2K, L).

**Fig. 3:**
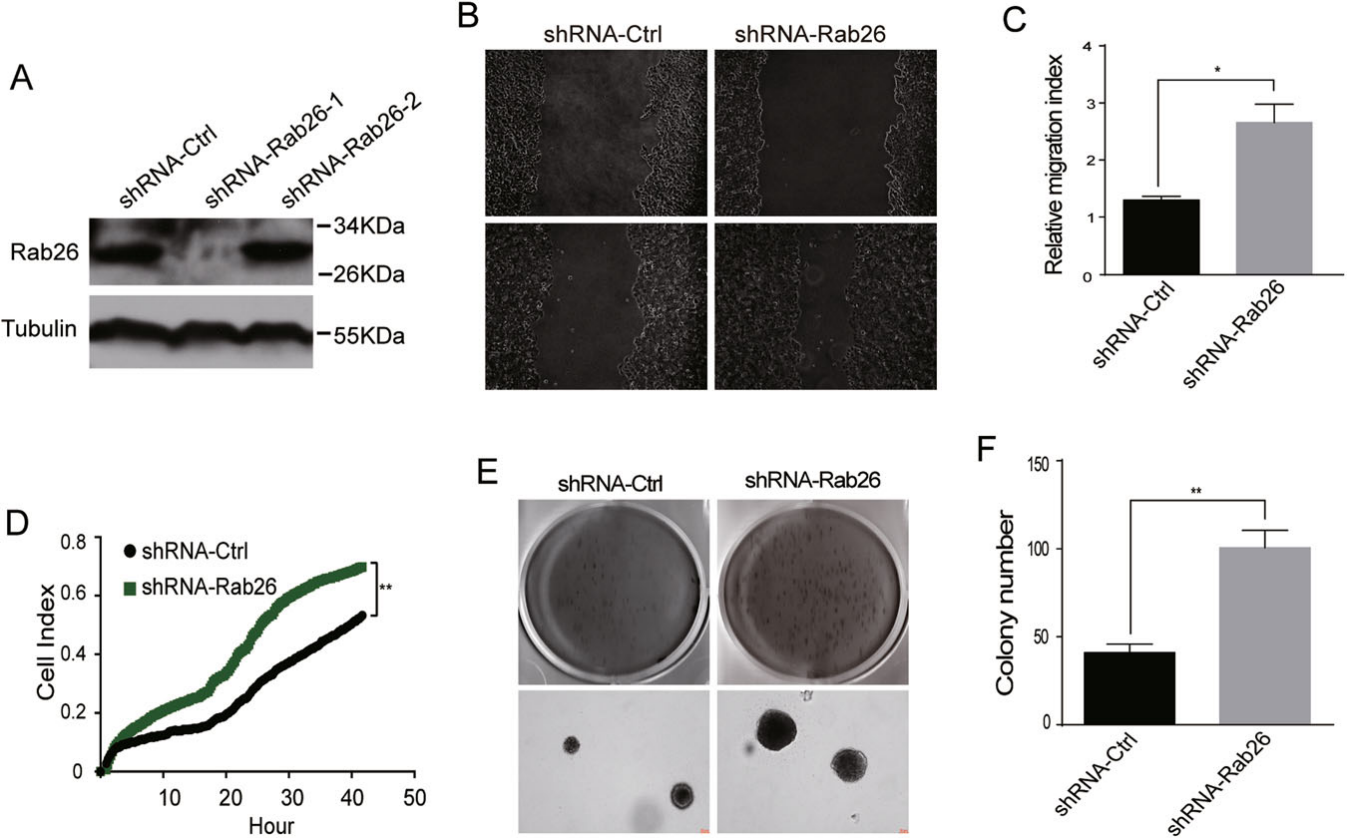
Rab26 knockdown promotes migration and invasion of breast cancer cells. **A** Western-blot showed shRNA-Rab26-1 efficiently depleted Rab26 in MCF7 cells. **B** Wound-healing assays showed Rab26 knockdown promotes migration of MCF7 cells. **C** Quantitative analysis of the wound area using imageJ software from three independent wound-healing experiments, *p < 0.05. **D** RTCA matrigel invasion assays showed Rab26 knockdown promotes invasion of MCF7 cells. **p < 0.01. **E** Double-layer soft agar experiments show that Rab26 knockdown promotes colony formation of MCF7 cells in soft agars. **F** Quantitative analysis of the results of **E** from three independent experiments. **p < 0.01.

To confirm the role of Rab26 in regulating migration/invasion of breast cancer cells, we tried to deplete Rab26 through lentivirus-mediated shRNA expression system in lower invasive and Rab26 higher-expressed MCF7 cells (Fig. 3A, shRNA-Rab26-1 was used in the following experiments as it efficiently depletes Rab26). We examined the migration/invasion capabilities of MCF7 cells infected with lentivirus expressing shRNA-Rab26. The results showed that Rab26 knockdown promotes the migration of lower-invasive MCF7 cells in wound-healing assay (Fig. 3B, C). RTCA instrument, which detects the cell index which increases with the number of cells migrating into the lower wells from the upper chamber in Matrigel assays, was used to examine the invasion of MCF7 cells, the data revealed that Rab26 knockdown greatly enhances the invasion of MCF7 cells (Fig. 3D). Additionally, Rab26 knockdown increases the levels of MMP2/MMP9 (Fig. S1D). The result that depletion of Rab26 promoted invasion of lower-invasive MCF7 cells was confirmed by soft-agar colony formation assay (Fig. 3E, F).

Additional experiments demonstrated that depletion of Rab26 does not influence cell proliferation (Fig. S1E, F). Taken together, Rab26 inhibits the migration and invasion of higher-invasive breast cancer cells, and Rab26 depletion conversely promotes the migration and invasion of lower-invasive breast cancer cells. These results suggest that Rab26 plays an important role in suppressing the migration and invasion of breast cancer cells.

### Rab26 downregulates the protein level of the phosphorylated Src in breast cancer cells

**Fig. 4:**
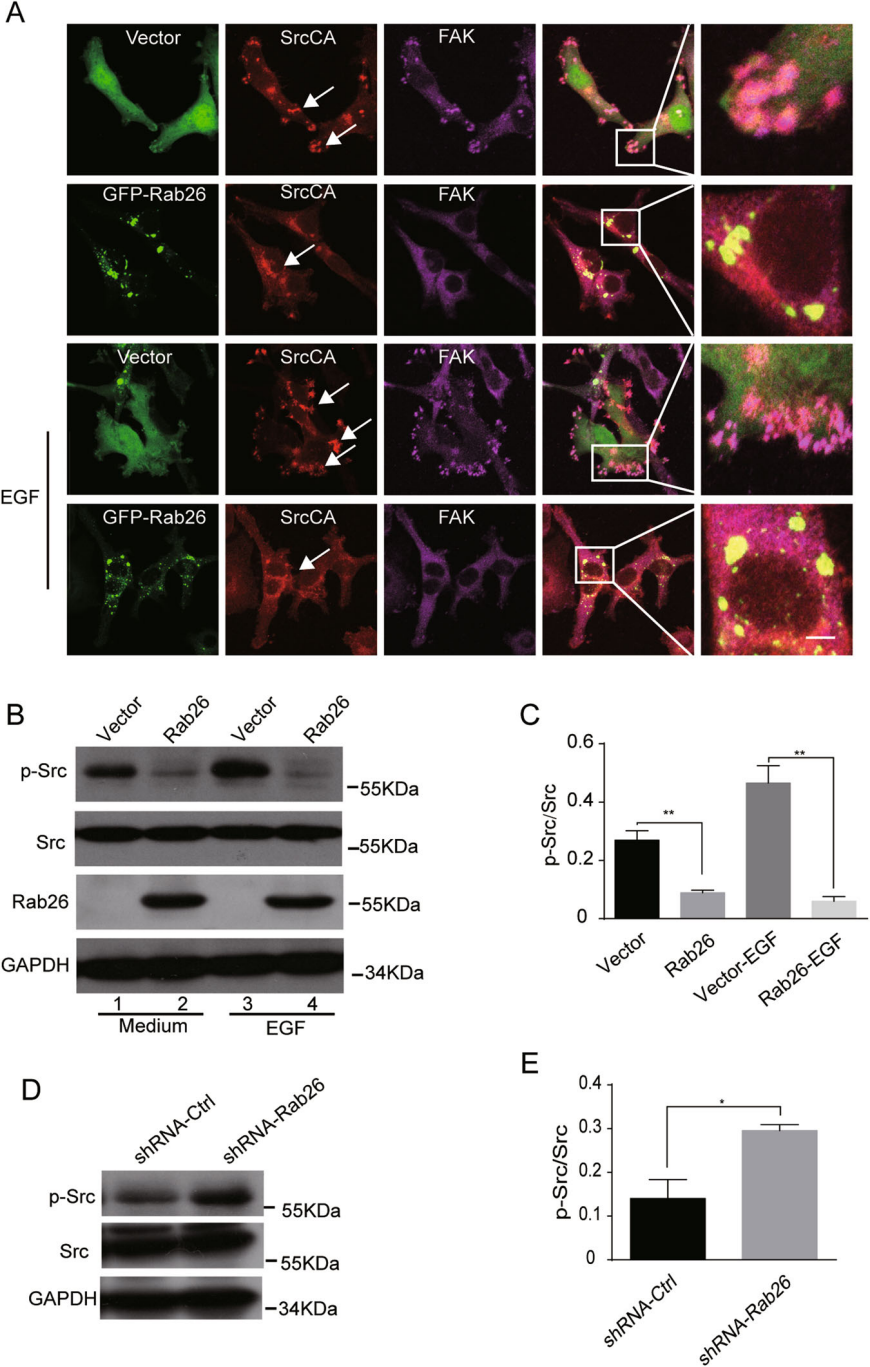
Rab26 downregulates the protein level of the active Src in breast cancer cells. **A** MDA-MB-231 cells were co-transfected with GFP-Rab26 and pUSEamp-SrcCA; the results showed that Rab26 alters the intracellular localization of SrcCA under normal condition or EGF treatment. Bar = 20 μm. **B** Western blot demonstrated over-expression of Rab26 reduces phosphorylated Src in the presence of EGF (100 ng/ml) or not in MDA-MB-231 cells. **C** Quantitative analysis of the results of **B** from three independent experiments. **p < 0.01. **D** Western-blot demonstrated Rab26 knockdown increases the protein level of phosphorylated Src in MCF7 cells. **E** Quantitative analysis of the results of **D** from three independent experiments. *p < 0.05.

Rab26 mediates degradation of phosphorylated Src in lung endothelial cells^30^, suggesting that Rab26 may regulate the migration/invasion of breast cancer cells through mediating Src activity. Therefore, we next investigated how Rab26 regulates Src in breast cancer cells. Immunofluorescence microscopy revealed that Rab26 is associated with both the early endosomes (marked by EEA1) and late endosomes/lysosomes (marked by Lamp1) (Fig. S2A). SrcCA (constitutive active form) associates at the plasma membrane and induces focal adhesion structures in MDA-MB-231 cells (Fig. 4A), which is essential for cell migration^36,37^. When co-expressing Rab26 with SrcCA, it was observed that the Src-induced focal adhesion labeled by FAK was dramatically reduced, and Src is now predominantly associated with Rab26-containing endosomal structures (Fig. 4A), this phenomenon was verified under EGF stimulation (Fig. 4A). For comparison, Rab11 (which regulates receptor recycling) did not alter the focal adhesion association of SrcCA (Fig. S2B).

The activated Src is tyrosine-phosphorylated and contributes to the formation of focal adhesion. Since over-expression of Rab26 reduced focal adhesion association of Src, we examined whether Rab26 influences the level of phosphorylated Src. Western-blot experiments demonstrated that the amount of phosphorylated Src was significantly reduced under steady status or EGF stimulation condition in Rab26-transfected MDA-MB-231 cells (Fig. 4B, C), but there is no major change for the total amount of Src. Conversely, Rab26 knockdown upregulated the phosphorylated Src in MCF7 cells (Fig. 4D, E). We detected the phosphorylation of FAK, the expression of Rab26 also slightly affects phosphorylation of FAK (Fig. S2C), probably due to the alternation of p-Src level. Additional experiments were carried out to examine the phosphorylated Src level in different breast cancer cell lines. The results demonstrated that most of the higher-invasive cells such as MDA-MB-231, BT549, and HCC-1806 have higher level of the phosphorylated Src (Y416) (Fig. S3), consistent with lower level of Rab26 in these cell lines.

**Fig. 5:**
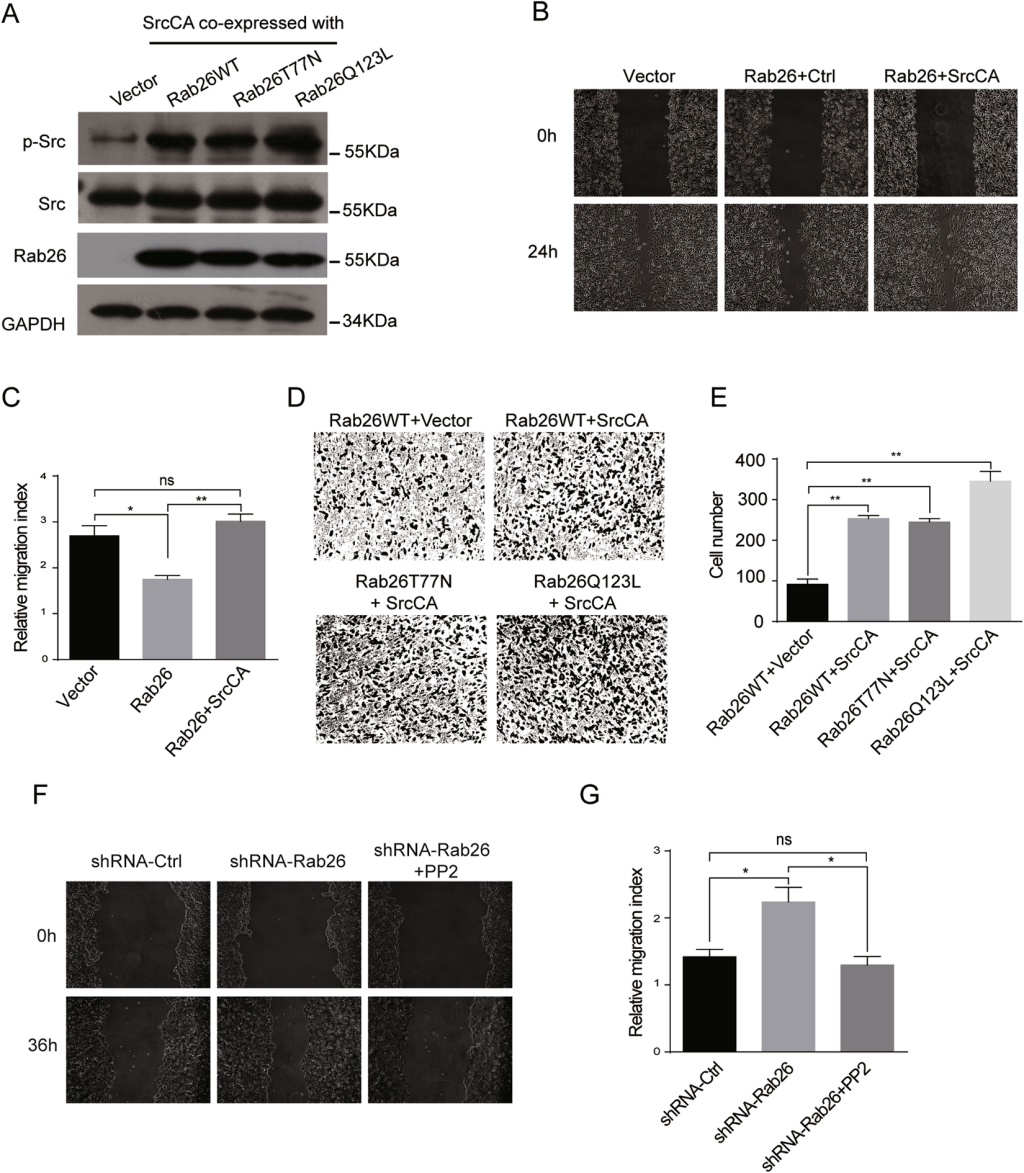
Expression of the active Src reverses the Rab26-induced inhibition for cell migration and invasion. **A** MDA-MB-231 cells stably expressing Rab26 were transfected with pUSEamp-SrcCA, western blot showing the expression of SrcCA. **B** Wound-healing assays showed SrcCA rescued migration of MDA-MB-231 cells expressing Rab26. **C** Quantitative analysis of the wound area using imageJ software from three independent wound-healing experiments, **p < 0.01, *p < 0.05. **D**, **E** Matrigel invasion assays showed SrcCA promotes invasion of MDA-MB-231 cells expressing Rab26. **p < 0.01. **F** Wound-healing assays showed inhibition of Src activity by PP2 inhibits migration of MCF7 cells with Rab26 depleted. **G** Quantitative analysis of the wound area using imageJ software from three independent wound-healing experiments, *p < 0.05.

To verify whether Rab26 inhibits the migration/invasion of breast cancer cells through downregulating phosphorylated Src, the phosphorylated Src was replenished in MDA-MB-231 cells stably expressing Rab26 by transfection with the constitutive active Src (SrcCA) (Fig. 5A). As expected, wound-healing assay revealed the migration ability of MDA-MB-231 cells expressing Rab26 was obviously restored upon transfection with SrcCA (Fig. 5B, C), also the invasive capability of cells expressing Rab26WT, T77N mutant, or Q123L mutant was significantly enhanced with expression of SrcCA (Fig. 5D, E). Since Rab26 knockdown prevents p-Src degradation, we used Src inhibitor PP2 to inhibit Src activity, then examined the migration of MCF7 cells with Rab26 depletion, the results demonstrated that PP2 inhibited the cell migration (Fig. 5F, G). These results indicate that Rab26 may modulate the Src activity through promoting degradation of the phosphorylated active Src in breast cancer cells under physiological condition, and consequently inhibits the migration/invasion of breast cancer cells.

### Rab26 mediates the autophagic degradation of phos-Src depending on the interaction with ATG16L1

**Fig. 6:**
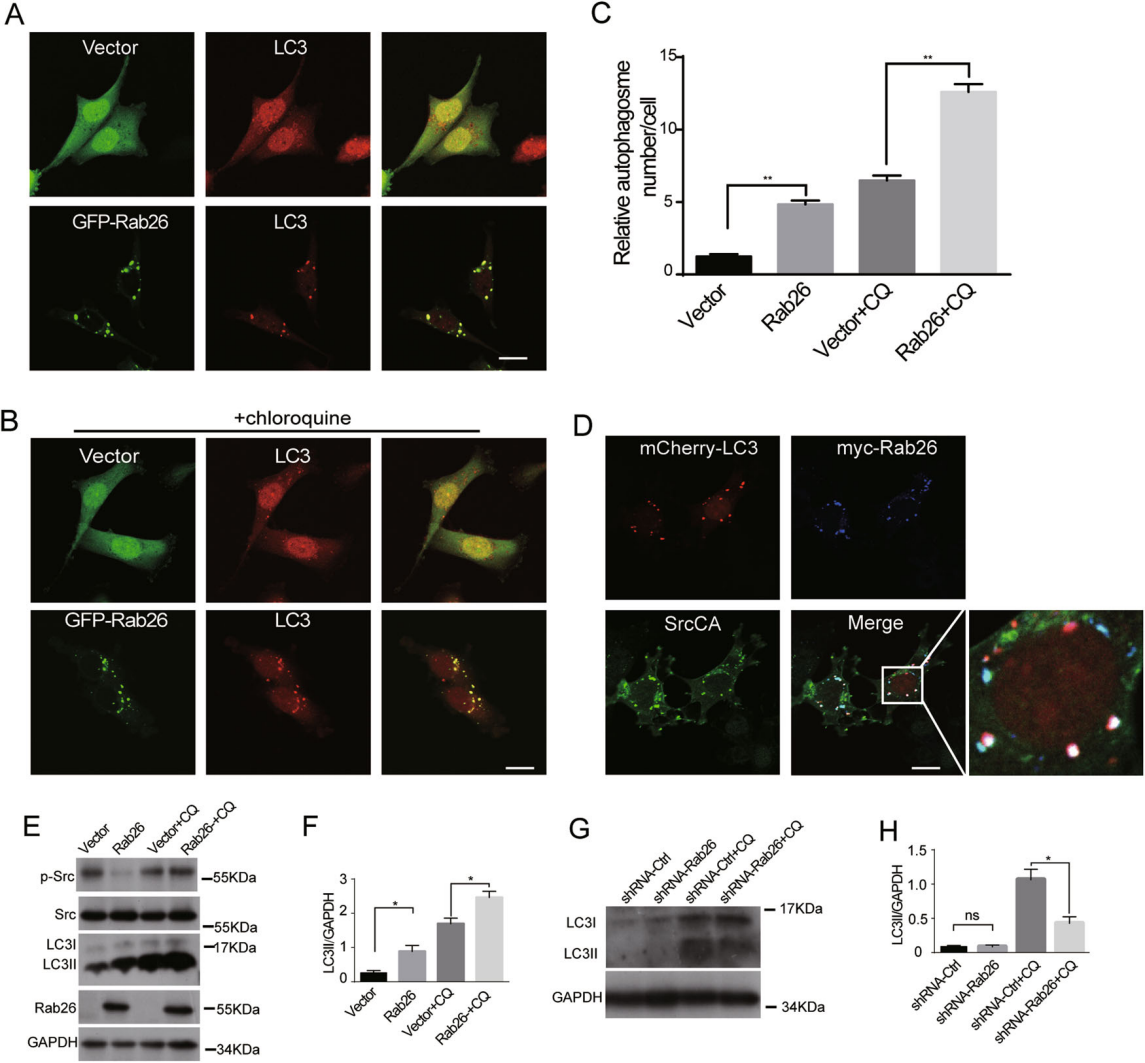
Rab26 induces the active Src targeting to autophagosome. **A** GFP-Rab26 is associated with autophagosomes marked by LC3 antibody in MDA-MB-231 cells. Bar = 20 μm. **B** Chloroquine (CQ) treatment increases the association of Rab26 with LC3. Bar = 20 μm. **C** Quantitative analysis by ImageJ software to reveal Rab26/LC3-positive compartments. **p < 0.01. **D** Over-expression of Rab26 induces SrcCA targeting to LC3 marked autophagosomes. Bar = 20 μm. **E** Western blot showed over-expression of Rab26 increases the level of LC3II. **F** Quantitative analysis of the results of **E** from three independent experiments. *p < 0.05. **G** Western blot showed Rab26 knockdown decreases the level of LC3II. **H** Quantitative analysis of the results of **G** from three independent experiments. *p < 0.05.

The previous investigations demonstrated that Rab26 is engaged in autophagic pathway^30^. Here MDA-MB-231 cells expressing Rab26 were used to verify Rab26 mediating autophagy process in breast cancer cells. Immunofluorescence microscopy showed that GFP-Rab26 is associated with autophagosomes marked by LC3 under normal culture condition or chloroquine treatment, which induces autophagy (Fig. 6A, B). The results revealed that Rab26 can significantly induces autophagosome formation (Fig. 6C). When co-transfected with Rab26 and SrcCA, it was observed that Rab26 induced SrcCA targeting to the autophagosomes marked by LC3 (Fig. 6D). Western-blot assay demonstrated that over-expression of Rab26 increases the protein levels of LC3II under both normal condition and chloroquine treatment (Fig. 6E, F). However, as chloroquine will inhibit lysosomal activity, Rab26 did not enhance the degradation of p-Src (phosphorylated Src) under chloroquine treatment (Fig. 6E, F). In addition, Rab26 knockdown decreases LC3II in MCF7 cells (Fig. 6G, H). These data indicate that Rab26 may enhance autophagy process and regulates the autophagic degradation of p-Src in breast cancer cells.

**Fig. 7:**
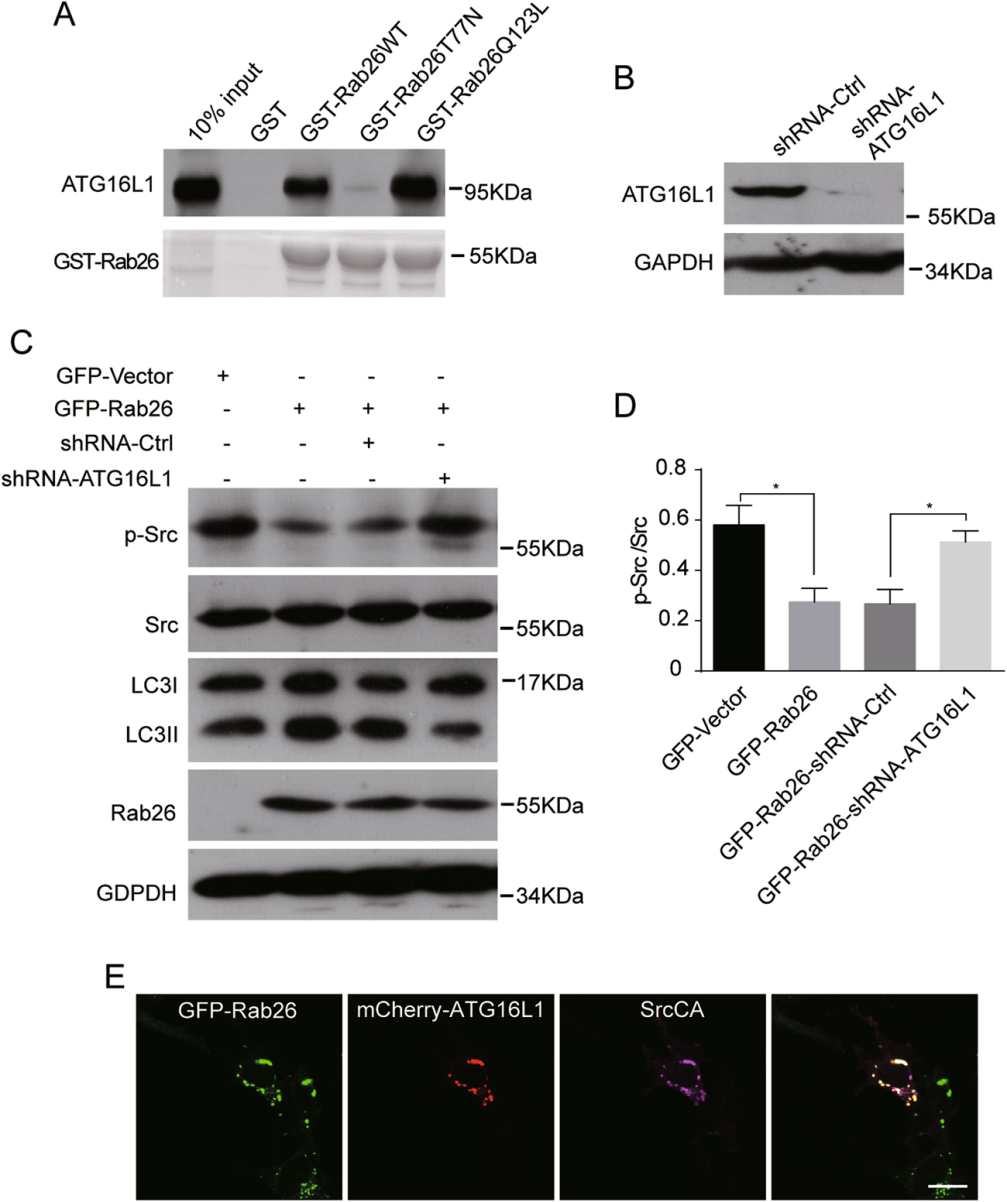
Rab26 mediates the autophagic degradation of phosphorylated Src depending on the interaction with ATG16L1. **A** GST pull-down and western-blot assay demonstrated that Rab26 interacts with ATG16L1. **B** Western blot showed ATG16L1 is efficiently depleted by the pSicoR-GFP lentiviral system in MDA-MB-231 cells. **C** Western blot demonstrated that ATG16L1 knockdown in Rab26 over-expressed cells restores phosphorylated Src levels. **D** Quantitative analysis of the results of **C** from three independent experiments. *p < 0.05. **E** GFP-Rab26 associates with mCherry-ATG16L1 at autophagosomes. Bar = 20 µm.

Rab26 participates in the autophagy process through interacting with ATG16L1 (refs. ^29,30^). In GST pull-down experiments, we found that Rab26 interacted with ATG16L1 in dependent of its guanine nucleotide-binding activity, as the interaction between Rab26T77N mutant and ATG16L1 was dramatically weakened (Fig. 7A). Next we examined whether the interaction of Rab26 with ATG16L1 is necessary for the Rab26-mediated degradation of p-Src through autophagy pathway. ATG16L1 was depleted by lentivirus-mediated shRNA expression system in MDA-MB-231 cells stably expressing Rab26 (Fig. 7B), and then the protein levels of p-Src and LC3 were detected by western blot. The results demonstrated that the protein levels of p-Src significantly increase and the levels of LC3II decrease (Fig. 7C, D), indicating that ATG16L1 knockdown inhibits autophagy and consequently rescues the Rab26-mediated degradation of p-Src. Again, immunofluorescence microscopy revealed that Rab26 induced SrcCA targeting to the ATG16L1-containing autophagosomes (Fig. 7E). Taken together, Rab26 mediated the autophagic degradation of phos-Src in dependent of the interaction with ATG16L.

### The N-terminal region of Rab26 is crucial for regulating migration/invasion of breast cancer cells

**Fig. 8:**
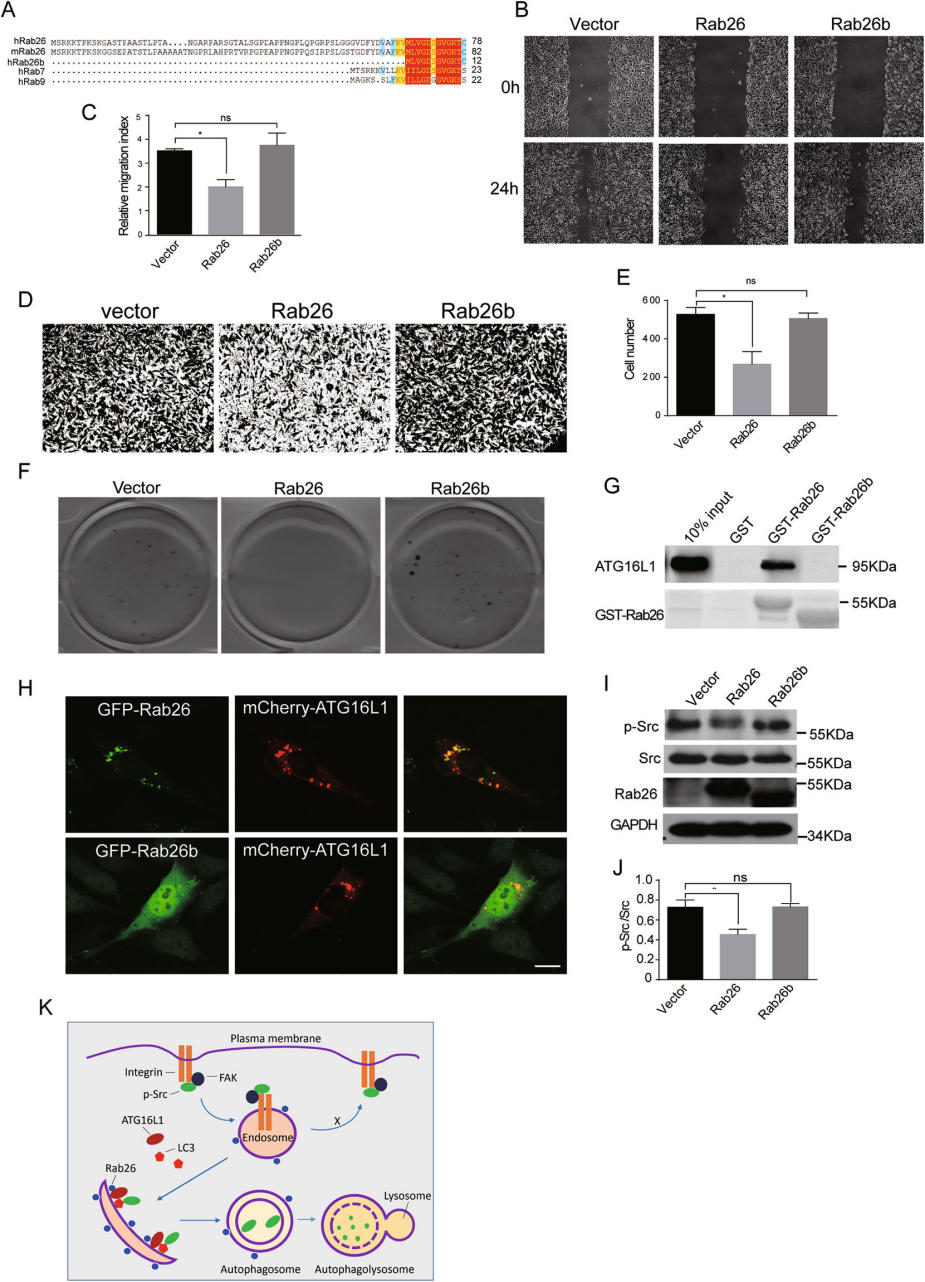
The N-terminal region of Rab26 is crucial for regulating migration/invasion of breast cancer cells. **A** Rab26 has an extra N-terminal extension in amino-acid sequence compared with other Rab proteins. **B** Wound-healing assays showed over-expression of Rab26b (which does not contain the N-terminal extension) did not inhibit migration of MDA-MB-231 cells. **C** Quantitative analysis of the wound area using imageJ software from three independent wound-healing experiments, *p < 0.05. **D** Matrigel transwell invasion assay showed over-expression of Rab26b does not inhibit invasion of MDA-MB-231 cells. **E** Quantitative analysis of **C** from three independent experiments, *p < 0.05. **F** Double-layer soft-agar experiments showed that over-expression of Rab26b does not inhibit colony formation of MDA-MB-231 cells in soft agar. **G** Rab26b does not interact with ATG16L1 in GST pull-down assay. **H** GFP-Rab26b is distributed in cytosol, not associating with mCherry-ATG16L1 in MDA-MB-231 cells, bar = 20 μm. **I** Rab26b does not induce the degradation of phosphorylated Src in western-blot experiments. **J** Quantitative analysis of the results of **I** from three independent experiments, *p < 0.05. **K** A model for Rab26 inhibiting cell migration/invasion through mediating autophagic degradation of phosphorylated Src. In this model, Rab26 associates with endosomes and recruits ATG16L1 to generate autophagophore, and subsequent LC3 to autophagosomal isolate membrane, LC3 then recruits the active Src (p-Src). After autophagosome maturation, it fuses with lysosome to degrade p-Src. The degradation of the active Src may inhibit formation or turnover of focal adhesion, consequently inhibiting cell migration and invasion.

Compared with other Rab proteins, Rab26 has an extra N-terminal extension in amino-acid sequence. We found that there are two transcription variants in GeneBank encoding two Rab26 isoforms (accession No. NM_014353.5 and NM_001308053.1), the longer one with 256aa was referred to as Rab26 and the other one lacking 66aa N-terminal extension sequence here was referred to as Rab26b (Fig. 8A). We investigated the effects of over-expressing Rab26b on migration and invasion of breast cancer cells. Wound-healing assay revealed that, unlike Rab26, over-expression of Rab26b did not inhibit the migration of MDA-MB-231 cells (Fig. 8B, C). Similarly, over-expression of Rab26b neither inhibits the cell invasion of MDA-MB-231 cells in matrigel invasion experiment (Fig. 8D, E) nor inhibits colony formation in soft-agar assay (Fig. 8F). These results suggest that Rab26b has different role in breast cancer cells as compared with Rab26.

Mechanistically, Rab26b is not able to interact with ATG16L1 in GST pull-down assay (Fig. 8G), suggesting the N-terminal extended sequence in Rab26 is essential for the interaction with ATG16L1. In addition, Rab26b is distributed mainly in the cytoplasm, not co-localizing with ATG16L1 (Fig. 8H). Further examination of the effects of Rab26b on Src demonstrated that over-expression of Rab26b did not influence the protein level of phosphorylated Src (Fig. 8I, J), suggesting Rab26b does not mediate the autophagic degradation of p-Src. Together with the above results, Rab26 and Rab26b have diverse functions in cancer cells, and Rab26 inhibits the migration and invasion of breast cancer cells through mediating the autophagic degradation of p-Src by interacting with ATG16L1 through its N-terminal region.

## Discussion

Many Rab proteins are proved to regulate the motility and invasion of cancer cells^38,39^. Rab26 regulates the degradation of phosphorylated Src and the trafficking of cell surface receptors^30,31^, suggesting a potential role of Rab26 in regulating cancer cell migration and invasion. There is evidence that SNRPB may promote the tumorigenic potential of NSCLC by regulating Rab26 expression; however, little is known about how Rab26 functions in cancer cells. In this study, we found that the highly aggressive breast cancer cells have a lower level of Rab26 protein, and over-expression of Rab26 in these cells inhibits the migration and invasion; on the other hand, Rab26 knockdown in less aggressive breast cancer cells promotes the invasion and migration. Mechanistically, our results uncover that Rab26 regulates the autophagic degradation of phosphorylated Src to inhibit the invasion and migration of breast cancer cells, indicating that Rab26 is a novel tumor suppressor for breast cancer.

Rab proteins regulate the endocytosis and recycling of surface receptors such as integrin and EGFR to sustain signaling for migration/invasion and proliferation of cancer cells^40,41^, or mediate sorting of surface MMP protein to modulate the motility of cancer cells^42^; in these cases, Rab proteins usually promote cancer development. In this study, we found Rab26 inhibits migration/invasion through mediating the autophagic degradation of active Src, suggesting a unique function of Rab26 and a different regulatory mechanism from other Rab proteins in breast cancer cells.

Expression of Rab26 protects the adheren junctional integrity in acute lung injury, while loss of Rab26 results in CDH5/VE-cadherin internalization, thereby weakening the integrity of adheren junction in pulmonary microvascular endothelial cells^30^; similarly, expression of Rab26 may keep the endothelial/epithelial property of cancer cells, and depletion of Rab26 may enhance the motility of cancer cells. Our findings demonstrated that Rab26 harnesses the same mechanism as that in non-cancer cells to regulate migration and invasion of breast cancer cells, in that Rab26 promotes the autophagic targeting and degradation of p-Src through interacting with ATG16L1.

Src is a key factor for focal adhesion, involved in migration and invasion of many types of cancer cells^3^. Integrin adhesion to extracellular matrix triggers downstream FAK activation, then FAK recruits Src to bind to integrin, in turn enhancing FAK activity^1,43^. The trafficking of Src to the focal adhesion depends on endosome-mediated trafficking pathway, which regulated by Rho family proteins and actin cytoskeleton^44,45^. Src also mediates the phosphorylation of Rab7 and Rab34 (refs. ^19,46^), suggesting Src may regulate endocytic trafficking reciprocally.

Our results strengthen that another trafficking pathway of Src, namely autophagic degradation of Src regulated by Rab26 in breast cancer cells, which has been described in pulmonary endothelial cells^30^. Cargo selected for autophagic degradation depends on the ubiquitination signals and specific receptors, which determined by the diverse upstream signaling pathways; therefore, only specific cargo proteins go for autophagic degradation under specific conditions. It is proved that only p-Src is ubiquitinated by c-Cbl, and consequently recognized by LC3 to target to autophagosome^47^. Therefore, Rab26 only mediates the degradation of p-Src, without affecting total Src.

Autophagic degradation may attenuate Src signaling to inhibit formation of focal adhesion, thereby inhibits cell migration and invasion. However, autophagic targeting of Src may promote cancer cell survival upon FAK depletion under autophagic conditions^47^. We found depletion of Rab26 has no significant effects on cell proliferation, indicating that Rab26-mediated autophagic degradation of Src not affecting cell viability, which is partially consistent with the previous investigation. In some cases, degradation of Src may enhance adhesion turn over to benefit migration^47^; however, our results indicates that a high expression of Rab26 may probably activate autophagy under normal conditions in breast cancer cells, thus constitutively mediate autophagic degradation of Src to maintain a lower level of Src, and consequently inhibit cell migration and invasion.

Our results demonstrated that Rab26 promotes autophagy in breast cancer cells, which probably due to enhance the recruitment of ATG16L1. ATG16L1 complexes with ATG12-ATG5 to regulate LC3 conjugating to phosphatidylethanolamine^48,49^. Src targets to autophasome by interaction with LC3 (refs. ^30,47^). Rab26 may dynamically associate with different endocytic compartments such as the early endosomes and late endo/lysosomes. During the endocytosis of procedure, the active Src goes to the endosomes, and autophagophore probably derive from the Rab26-containing early endosomes. Thus, we propose that Rab26 recruits ATG16L1, and subsequent LC3 to endosomes to generate autophagosome membrane, then LC3 recruits the active Src to autophagosome, and finally, autophagosome fuses with lysosome to degrade Src. The degradation of the active Src inhibits the FAK activation, consequently inhibiting cell migration and invasion (Fig. 8K).

Rab26 is the downstream target gene of transcript factor MIST1 (ref. ^27^). Over-expression of MIST1 reverses the EMT and reduces the tumorigenicity of pancreatic cancer cells^50^, which is consistent with our results that activation of Rab26 inhibits migration/invasion of cancer cells. Rab26 is specifically lower expressed in highly aggressive breast cancer lines such as MDA-MB-231 and BT549 (triple-negative breast cancer cell lines); however, we found Rab26b (probably a transcriptional variant of Rab26) possesses divergent function from Rab26, suggesting a potential transcriptional regulation of Rab26 expression in different cells, which deserves further investigations. Our findings provide a potential strategy by activating MIST1–Rab26 pathway for cancer therapy.

## Materials and methods

### Antibodies

Rabbit polyclonal antibodies for Rab26 (cat.14284-1-AP), LC3 (cat.14600-1-AP for western blot), mouse monoclonal antibodies (mAb) against GAPDH (cat.60004-1-Ig), FAK (cat.66258-1-Ig), and GFP (cat.66002-1-Ig) were purchased from Proteintech (Wuhan, China). Rabbit polyclonal antibody against TGN46 (cat.T7576) was from Sigma Aldrich (St Louis, MO, USA). mAb for FAK (pY397) (cat.611722) and EEA1 (cat.610456) were purchased from BD Biosciences (Palo Alto, CA). Rabbit polyclonal antibody for LC3 (cat.ab51520, for immunofluorescence staining) was from Abcam (Shanghai, China). Rabbit polyclonal antibody for Src (cat.2108), p-Src(Tyr416) (cat.6943), MMP2 (cat.40994), and MMP9 (cat.13667) were obtained from Cell Signalling Technology (Danvers, Massachusetts, USA). Rabbit polyclonal antibody for ATG16L1 (cat.D262704) was purchased from Sangon Biotech (Shanghai, China). mAb for Lamp1 were from Developmental Studies Hybridoma Bank (Iowa City, IA, USA). HRP-conjugated secondary antibodies, Texas red-conjugated and Cy5-conjugated secondary antibodies were obtained from Jackson Immuno Research (cat. 111-035-003, 115-025-003, 111-095-003, 111-175-144, West Grove, PA, USA).

### Expression plasmids

GFP-Rab26 (referred to as Rab26WT) expression plasmid was generated by cloning the coding region of cDNA for human Rab26 into pEGFP-C1 vector. The plasmids for Rab26b (a truncated form lacking the N-terminal 66aa), the dominant-negative mutant GFP-Rab26T77N and constitutive active mutant GFP-Rab26Q123L were generated by the PCR-directed mutagenesis approach. Similarly, the coding region for Rab26 and its mutants were subcloned into pGEX-4T-1 vector, pmCherry-C1, pDmyc-Vector or pEGFP-C1 vectors to generate the corresponding plasmids for expressing GST-/mCherry-/GFP-/myc-tagged protein, respectively. The coding region for ATG16L1 was retrieved from cDNA derived from MCF7 cells and subcloned into pEGFP-C1 or pmCherry-C1 vector to generate GFP-ATG16L1 or mCherry-ATG16L1 expression plasmids. mCherry-LC3 plasmid is from Dr. Wanjin Hong (Institute of Molecular and Cell Biology, Singapore). The plasmid for SrcCA (constitutive active mutant) was described^46^. All constructed plasmids were finally confirmed by DNA sequencing.

### Cell culture and transfection

Breast cancer cell lines were obtained from ATCC (American Type Culture Collection). MDA-MB-231, HS578t, MCF7, and 293t cells were cultured in Dulbecco’s modified Eagle’s medium (DMEM) containing 10% fetal bovine serum (FBS), and BT549, SK-Br-3, HCC-1806, and BT474 cells were cultured in RPMI-1640 medium containing 10% fetal bovine serum in a 5% CO_2_ incubator at 37 °C. For EGF (Pepro Tech, USA) treatment, Chloroquine (Sigma Aldrich, USA) treatment (CQ) or PP2 (Cayman, China) treatment, the MDA-MB-231/MCF7 cells were treated with EGF (100 ng/ml), CQ (20 µM), or PP2 (4 µM) for the indicated time under normal culture conditions. For cell transfection, cells were transfected with the indicated plasmids by using Lipofectamine2000 reagents according to the manufacturer’s instruction.

### pSicoR-mediated gene silencing and pCDH-mediated lentivirus expression system

Targeting sequences shRNA-Rab26-1 (5′-CCGGCTGCATGATTACGTTAA-3′), shRNA-Rab26-2 (5′-GCATTGACTTCCGGAACAAAG-3′), and shRNA-ATG16L1 (5′-GCCTGGAAGAATAACACTGAA-3′) were used for gene knockdown of Rab26 or ATG16L1 through lentiviral vector pSicoR-mediated gene knockdown system, respectively. The over-expression of Rab26 and Rab26b was achieved by pCDH-CMV-MCS-EF1-Puro vector-mediated lentivirus expression system. For virus preparation, 293t cells were transfected with lentiviral backbone and helper plasmids (pMD2.G, psPAX2) for 48 h and collected the culture media. The infection of target cell lines was treated with virus culture media overnight for over-expression or knockdown experiments. Western blot was used to verify the expression level of target protein.

### Western-blot analysis

Cells were lysed in lysis buffer (containing 20 mM HEPES, pH 7.4, 150 mM NaCl, 0.5% Triton X-100, and EDTA-free proteinase inhibitor cocktail (MedChemexpress, USA) and phosphatase inhibitor (Yeasen, China)) on ice. The lysates were spun down at 13,000*g* for 30 min at 4 °C. The resulted cell lysates were quantified using BCA kit (Thermo Scientific, USA). Proteins were resolved by SDS-PAGE and transferred to nitrocellulose membrane, and then blocked with 5% bovine serum albumin (BSA). The corresponding membranes were incubated with primary antibodies overnight at 4 °C, following the incubation with HRP-conjugated secondary antibodies. The bands were visualized by an ECL kit (Thermo Scientific, USA). The blots were quantified by analysis of the grayscale using imageJ software.

### Immunohistochemistry

The breast cancer tissues arrays (US Biomax, USA) on glass slides were subjected to detect the expression profile of Rab26 according to the manufacturer’s protocol. Briefly, after deparaffinization and rehydration of specimens, the tissues was performed antigen retrieval treatment, and blocked with 1% BSA. The array was incubated with Rab26 antibody followed by HRP-conjugated secondary antibody (ZSGB-BIO, China) and then DAB (3,3′-diaminobenzidine). Hematoxylin was used to label nuclear. The tissue samples were observed and analyzed under a microscope (Olympus BX53, Japan).

### Immunofluorescence microscopy analysis

Immunofluorescence staining was performed as described^19^. Briefly, cells seeded on cover glasses were washed with phosphate-buffered saline (PBS) and fixed with 4% paraformaldehyde for 30 min at 4 °C, and then permeabilized with 0.1% Triton X-100 for 15 min at room temperature. The cells were incubated with the primary antibody at room temperature for 1 h, followed by incubation with fluorescein-conjugated secondary antibody. The samples were observed and analyzed by confocal immunofluorescence microscopy (Carl Zeiss LSM5 EXITER laser, Zeiss, Jena, Germany).

### GST pull-down assay

293t transfected with the indicated plasmids were lysed with lysis buffer (containing 20 mM HEPES, pH 7.4, 150 mM NaCl, 0.5% Triton X-100, and EDTA-free proteinase inhibitor cocktail) on ice for 1 h. The lysates were centrifuged at 13,000*g* for 15 min at 4 °C. The supernatants were incubated with GST/GST-Rab26/Rab26b coupled to GST-Sepharose 4B resin (GE Healthcare, USA) at 4 °C for overnight. GST-Sepharose 4B resin were washed three times with lysis buffer containing different concentrations of NaCl (500, 300, 100 mM). The bound proteins were analyzed by Western-blot assay. GST fusion protein is stained with Coomassie Brilliant Blue (Sigma Aldrich, USA).

### Wound-healing experiment and matrigel invasion experiment

For wound-healing assay, cells grow in six-well plates to about 80–90% confluence. The cell monolayer was scratched using a yellow tip to generate wound under aseptically conditions. The detached cells were washed away with PBS, and then the fresh media were added to allow cells grow to heal the wound gap. Wound healing were observed under a microscope.

For the invasion experiment, cells were seeded in upper transwell chambers of a 24-well plate which was coated with Matrigel (Coring, USA), and the appropriate amount of cells were resuspended in serum-free medium and placed in the upper chambers. The cells will invade into the lower wells containing 800 µl medium containing 10% FBS. After invasion for 24 h, cells on the top of the membrane were removed, and then fixed with 4% paraformaldehyde and stained with 0.1% crystal violet. The invaded cells were observed under a microscope. Quantitative analysis was processed through ImageJ/GraphPad Prism software. RTCA (real-time cell analysis) was applied for lower-invasive MCF7 cells using a xCELLigence RTCA (ACEA Biosciences, USA) instrument according to the manufacturer’s instruction.

### Soft-agar assay

In all, 0.6% agarose (Solarbio, China) in DMEM medium was added to the six-well plate to make a lower layer agarose gel. Five thousand cells of each groups were resuspended in 2× DMEM medium and mixed with an equal volume of 0.7% agarose, and added on the lower gel to generate 0.35% upper layer soft agar. The double-layer gel was covered with DMEM. After 2 weeks, the cell colonies were counted.

### Metastasis experiments in nude mice

Metastasis experiments in nude mice were carried out as described with minor modifications^19^. Cells over-expressing Rab26 or control vector (1.0 × 10^6^) were injected into nude mice (each group contains five female mice of 5-week-old with the body weight about 18–22 g) through the tail vein, nude mice were sacrificed after 5 weeks, and lung tissues were directly removed to observe the number of tumors through HE staining. For HE staining, lung tissues were embedded in paraffin and processed for HE staining as described^51^. Briefly, tissue sections on glass slides were rehydrated with xylene and alcohol, and counterstained with hematoxylin and eosin to label nuclear and cytoplasm, respectively. Tissue samples were observed under a microscope (Microdigital section scanning system Motic VM1, China).

All animal experimental operations were carried out in compliance with the guidelines for the care of laboratory animals in strict compliance with the regulations of Institutional Animal Ethics Committee of Xiamen University.

### Statistical analysis

Prism 6.0 was utilized to perform quantitative analysis of the results from three independent experiments. In general, two-tailed Student’s *t*-tests were performed to indicate statistical significance; bar graph represents quantification as mean ± SEM of three independent experiments (*n* = 3), The mean values of two groups were considered significant difference at *p < 0.05, **p < 0.01, and “ns” means no significant difference of two groups. For animal experiments, the non-blind method evaluation and random grouping method was used for the grouping of nude mice.

## Supplementary information

Supplemental Table S1

supplemental figure legends

Figure S1

Figure S2

Figure S3

Related Manuscript File

## Data Availability

Data and resource are available from the corresponding authors.
