# Supplementary material for: Rab26 suppresses migration and invasion of breast cancer cells through mediating autophagic degradation of phosphorylated Src
Source: Cell Death Dis. 2021 Mar 17;12(4):284. doi: 10.1038/s41419-021-03561-7 (PMC7969620; doi:10.1038/s41419-021-03561-7)
Supplement: Supplementary file 1 — Supplemental Table S1 [file 41419_2021_3561_MOESM1_ESM.docx]

Table S1. Immunohistochemical analysis of breast cancer tissue array

| No. | Age | Sex | Organ | Pathology diagnosis | Grade | | TNM | Stage | Tissue ID. | Type | Expression | |
| --- | --- | --- | --- | --- | --- | --- | --- | --- | --- | --- | --- | --- |
| A1 | 14 | F | Breast | Breast tissue | - | - | | - | Fmg03N001 | Normal | | ++ |
| A2 | 15 | F | Breast | Breast tissue | - | - | | - | Fmg06N002 | Normal | | +++ |
| A3 | 18 | F | Breast | Breast tissue  (fibrous and fatty tissue) | - | - | | - | Fmg06N010 | Normal | | + |
| A4 | 21 | F | Breast | Breast tissue  (ductal ectasia) | - | - | | - | Fmg06N023 | Normal | | ++ |
| A5 | 21 | F | Breast | Breast tissue | - | - | | - | Fmg06N024 | Normal | | +++ |
| A6 | 35 | F | Breast | Breast tissue  (adenosis) | - | - | | - | Fmg07N011 | Normal | | + |
| A7 | 19 | F | Breast | Breast tissue | - | - | | - | Fmg07N013 | Normal | | ++ |
| A8 | 39 | F | Breast | Adjacent normal breast tissue | - | - | | - | Fmg010509 | NAT | | + |
| A9 | 48 | F | Breast | Adjacent normal breast tissue | - | - | | - | Fmg010213 | NAT | | ++ |
| A10 | 47 | F | Breast | Adjacent normal breast tissue (fibrous and fatty tissue) | - | - | | - | Fmg010230 | NAT | | + |
| B1 | 30 | F | Breast | Fibroadenoma | - | - | | - | Fmg010006 | Benign | | +++ |
| B2 | 44 | F | Breast | Fibroadenoma | - | - | | - | Fmg010224 | Benign | | +++ |
| B3 | 23 | F | Breast | Fibroadenoma | - | - | | - | Fmg010031 | Benign | | +++ |
| B4 | 25 | F | Breast | Fibroadenoma | - | - | | - | Fmg010226 | Benign | | +++ |
| B5 | 19 | F | Breast | Fibroadenoma | - | - | | - | Fmg010034 | Benign | | + |
| B6 | 21 | F | Breast | Fibroadenoma | - | - | | - | Fmg010228 | Benign | | ++ |
| B7 | 42 | F | Breast | Fibroadenoma | - | - | | - | Fmg010036 | Benign | | + |
| B8 | 34 | F | Breast | Fibroadenoma | - | - | | - | Fmg010041 | Benign | | ++ |
| B9 | 29 | F | Breast | Fibroadenoma | - | - | | - | Fmg010044 | Benign | | + |
| B10 | 23 | F | Breast | Fibroadenoma | - | - | | - | Fmg010045 | Benign | | + |
| C1 | 25 | F | Breast | Fibroadenoma | - | - | | - | Fmg010046 | Benign | | +++ |
| C2 | 23 | F | Breast | Fibroadenoma | - | - | | - | Fmg010048 | Benign | | ++ |
| C3 | 32 | F | Breast | Fibroadenoma | - | - | | - | Fmg010050 | Benign | | +++ |
| C4 | 46 | F | Breast | Fibroadenoma | - | - | | - | Fmg010061 | Benign | | + |
| C5 | 57 | F | Breast | Fibroadenoma | - | - | | - | Fmg010227 | Benign | | + |
| C6 | 32 | F | Breast | Fibroadenoma | - | - | | - | Fmg010066 | Benign | | ++ |
| C7 | 33 | F | Breast | Fibroadenoma | - | - | | - | Fmg010067 | Benign | | ++ |
| C8 | 54 | F | Breast | Fibroadenoma | - | - | | - | Fmg010183 | Benign | | ++ |
| C9 | 38 | F | Breast | Fibroadenoma | - | - | | - | Fmg010212 | Benign | | ++ |
| C10 | 29 | F | Breast | Fibroadenoma | - | - | | - | Fmg010222 | Benign | | ++ |
| D1 | 49 | F | Breast | Invasive lobular carcinoma | - | T3N0M0 | | IIIA | Fmg031293 | Malignant | | ++ |
| D2 | 53 | F | Breast | Invasive lobular carcinoma | - | T4N1M0 | | IIIB | Fmg030762 | Malignant | | + |
| D3 | 70 | F | Breast | Invasive lobular carcinoma | - | T2N2M0 | | IIIA | Fmg032386 | Malignant | | + |
| D4 | 49 | F | Breast | Invasive lobular carcinoma | - | T3N1M0 | | IIIA | Fmg040140 | Malignant | | + |
| D5 | 50 | F | Breast | Invasive lobular carcinoma | - | T2N0M0 | | IIA | Fmg020021 | Malignant | | + |
| D6 | 59 | F | Breast | Invasive lobular carcinoma | - | T4M0N0 | | IIIB | Fmg020022 | Malignant | | + |
| D7 | 38 | F | Breast | Invasive lobular carcinoma (chronic inflammation of breast tissue) | * | T2N1M0 | | IIB | Fmg010232 | Malignant | | ++ |
| D8 | 38 | F | Breast | Invasive lobular carcinoma | - | T2N0M0 | | IIA | Fmg020138 | Malignant | | _ |
| D9 | 52 | F | Breast | Invasive lobular carcinoma | - | T2N0M0 | | IIA | Fmg020360 | Malignant | | _ |
| D10 | 52 | F | Breast | Invasive lobular carcinoma | - | T4N0M0 | | IIIB | Fmg020350 | Malignant | | _ |
| E1 | 46 | F | Breast | Invasive ductal carcinoma | 2 | T2N1M0 | | IIB | Fmg031129 | Malignant | | ++ |
| E2 | 40 | F | Breast | Invasive ductal carcinoma | 1 | T4N2M0 | | IIIB | Fmg041099 | Malignant | | + |
| E3 | 57 | F | Breast | Invasive ductal carcinoma | 1 | T3N1M0 | | IIIA | Fmg031077 | Malignant | | + |
| E4 | 28 | F | Breast | Invasive ductal carcinoma | 2 | T3N2M0 | | IIIA | Fmg010628 | Malignant | | + |
| E5 | 52 | F | Breast | Invasive ductal carcinoma | 2 | T4N1M0 | | IIIB | Fmg030226 | Malignant | | + |
| E6 | 39 | F | Breast | Invasive ductal carcinoma | 2 | T4N1M0 | | IIIB | Fmg030230 | Malignant | | _ |
| E7 | 51 | F | Breast | Invasive ductal carcinoma | 3 | T2N1M0 | | IIB | Fmg030243 | Malignant | | _ |
| E8 | 41 | F | Breast | Invasive ductal carcinoma | 2 | T2N2M0 | | IIIA | Fmg030342 | Malignant | | _ |
| E9 | 46 | F | Breast | Invasive ductal carcinoma | 2 | T4N2M0 | | IIIB | Fmg030516 | Malignant | | + |
| E10 | 61 | F | Breast | Invasive ductal carcinoma | 2 | T2N1M0 | | IIB | Fmg030088 | Malignant | | _ |
| F1 | 31 | F | Breast | Invasive ductal carcinoma | 2 | T3N1M0 | | IIIA | Fmg050134 | Malignant | | + |
| F2 | 50 | F | Breast | Invasive ductal carcinoma | 2 | T2N2M0 | | IIIA | Fmg030785 | Malignant | | + |
| F3 | 38 | F | Breast | Invasive ductal carcinoma | 2 | T2N1M0 | | IIB | Fmg030137 | Malignant | | + |
| F4 | 45 | F | Breast | Invasive ductal carcinoma | 2 | T4N1M0 | | IIIB | Fmg030834 | Malignant | | + |
| F5 | 58 | F | Breast | Invasive ductal carcinoma | 2 | T4N1M0 | | IIIB | Fmg030897 | Malignant | | + |
| F6 | 57 | F | Breast | Invasive ductal carcinoma | 1 | T2N1M0 | | IIB | Fmg030935 | Malignant | | + |
| F7 | 49 | F | Breast | Invasive ductal carcinoma | 2 | T1N1M0 | | IIA | Fmg030960 | Malignant | | + |
| F8 | 48 | F | Breast | Invasive ductal carcinoma | 2 | T2N1M0 | | IIB | Fmg031009 | Malignant | | _ |
| F9 | 57 | F | Breast | Invasive ductal carcinoma | 2 | T4N2M0 | | IIIB | Fmg030100 | Malignant | | _ |
| F10 | 42 | F | Breast | Invasive ductal carcinoma | 2 | T4N1M0 | | IIIB | Fmg031176 | Malignant | | + |
| G1 | 42 | F | Breast | Invasive ductal carcinoma | 2 | T4N2M0 | | IIIB | Fmg031305 | Malignant | | + |
| G2 | 51 | F | Breast | Invasive ductal carcinoma | 2 | T3N2M0 | | IIIA | Fmg031322 | Malignant | | ++ |
| G3 | 47 | F | Breast | Invasive ductal carcinoma | 2 | T3N2M0 | | IIIA | Fmg031624 | Malignant | | +++ |
| G4 | 36 | F | Breast | Invasive ductal carcinoma | 3 | T2N2M0 | | IIIA | Fmg031675 | Malignant | | + |
| G5 | 37 | F | Breast | Invasive ductal carcinoma | 2 | T3N1M0 | | IIIA | Fmg032005 | Malignant | | + |
| G6 | 36 | F | Breast | Invasive ductal carcinoma | 2 | T2N1M0 | | IIB | Fmg040045 | Malignant | | + |
| G7 | 58 | F | Breast | Invasive ductal carcinoma | 2 | T3N2M0 | | IIIA | Fmg040242 | Malignant | | + |
| G8 | 50 | F | Breast | Invasive ductal carcinoma | 2 | T4N1M0 | | IIIB | Fmg040311 | Malignant | | + |
| G9 | 45 | F | Breast | Invasive ductal carcinoma | 2 | T3N1M0 | | IIIA | Fmg040344 | Malignant | | _ |
| G10 | 53 | F | Breast | Invasive ductal carcinoma | 2 | T2N1M0 | | IIB | Fmg030111 | Malignant | | + |
| H1 | 53 | F | Lymp-hnode | Metastatic invasive ductal carcinoma | 2 | - | | - | Fmg032458 | Metastasis | | + |
| H2 | 46 | F | Lymp-hnode | Metastatic invasive ductal carcinoma of No. 41 | 2 | - | | - | Fmg031129 | Metastasis | | +++ |
| H3 | 61 | F | Lymp-hnode | Metastatic invasive ductal carcinoma of No. 50 | 2 | - | | - | Fmg030088 | Metastasis | | + |
| H4 | 57 | F | Lymp-hnode | Metastatic invasive ductal carcinoma of No. 59 | 2 | - | | - | Fmg030100 | Metastasis | | + |
| H5 | 53 | F | Lymp-hnode | Metastatic invasive ductal carcinoma of No. 70 | 3 | - | | - | Fmg030111 | Metastasis | | _ |
| H6 | 38 | F | Lymp-hnode | Metastatic invasive ductal carcinoma of No. 53 | 3 | - | | - | Fmg030137 | Metastasis | | _ |
| H7 | 52 | F | Lymp-hnode | Metastatic invasive ductal carcinoma of No. 45 | 2 | - | | - | Fmg030226 | Metastasis | | + |
| H8 | 39 | F | Lymp-hnode | Metastatic invasive ductal carcinoma of No. 46 | 2 | - | | - | Fmg030230 | Metastasis | | _ |
| H9 | 51 | F | Lymp-hnode | Metastatic invasive ductal carcinoma of No. 47 | 3 | - | | - | Fmg030243 | Metastasis | | _ |
| H10 | 41 | F | Lymp-hnode | Metastatic invasive ductal carcinoma of No. 48 | 3 | - | | - | Fmg030342 | Metastasis | | _ |

* represented by the degree of Rab26 staining."-" no expression; "+": lower expression; "++" moderate expression, "+++" high expression
