# Supplementary material for: Rab26 suppresses migration and invasion of breast cancer cells through mediating autophagic degradation of phosphorylated Src
Source: Cell Death Dis. 2021 Mar 17;12(4):284. doi: 10.1038/s41419-021-03561-7 (PMC7969620; doi:10.1038/s41419-021-03561-7)
Supplement: Supplementary file 2 — supplemental figure legends [file 41419_2021_3561_MOESM2_ESM.docx]

**Rab26 suppresses migration and invasion of breast cancer cells through mediating autophagic degradation of phosphorylated Src**

Huiying Liu^1*^, Yuxia Zhou^2*^, Hantian Qiu^1^, Ruijuan Zhuang^1^, Yang Han^1^, Xiaoqing Liu^1^, Xi Qiu^1^, Ziyan Wang^1^, Liju Xu^1^, Ran Tan^1^, Wanjin Hong^1,3^, Tuanlao Wang^1#^

**Supplementary figures**

**Fig.S1** A. The expression of Rab26 transcript from RT-PCR in all 8 breast cancer cell lines compared with the normal breast tissue cell line MCF10A. B. Western-blot to verify the expression plasmids of Rab26 in MDA-MB-231 cells using GFP antibody. C. Western-blot to demonstrate the effects of over-expression of Rab26 on MMP2/MMP9 in MDA-MB-231 cells. D. Western-blot to demonstrate the effects of Rab26 knockdown on MMP2/MMP9 in MCF7 cells. E. MTT assay to demonstrate the effects of Rab26 knockdown on cell proliferation of MCF7 Cells. F. The effects of Rab26 knockdown on cell growth and proliferation in 6-well plate.

**Fig.S2** A. Immunofluorescence microscopy to reveal the subcellular location of GFP-Rab26 using the endosomal markers (EEA1 for early endosomes, Lamp1 for late endosomes/lysosomes) in MDA-MB-231 cells. Bar=20μm. B. The effects of over-expression of Rab11 on the focal adhesion association of SrcCA in the presence of EGF (100ng/ml) or not in the MDA-MB-231 cells, Bar=20μm. C. Western-blot to demonstrate the effects of over-expression of Rab26 on the phosphorylation of FAK (pY397) in MDA-MB-231 cells.

**Fig.S3** Western-blot to show the levels of phosphorylated Src(Tyr416) in different breast cancer cell lines.
