# Supplementary figures and images for: Rab26 suppresses migration and invasion of breast cancer cells through mediating autophagic degradation of phosphorylated Src
Source: Cell Death Dis. 2021 Mar 17;12(4):284. doi: 10.1038/s41419-021-03561-7 (PMC7969620; doi:10.1038/s41419-021-03561-7)

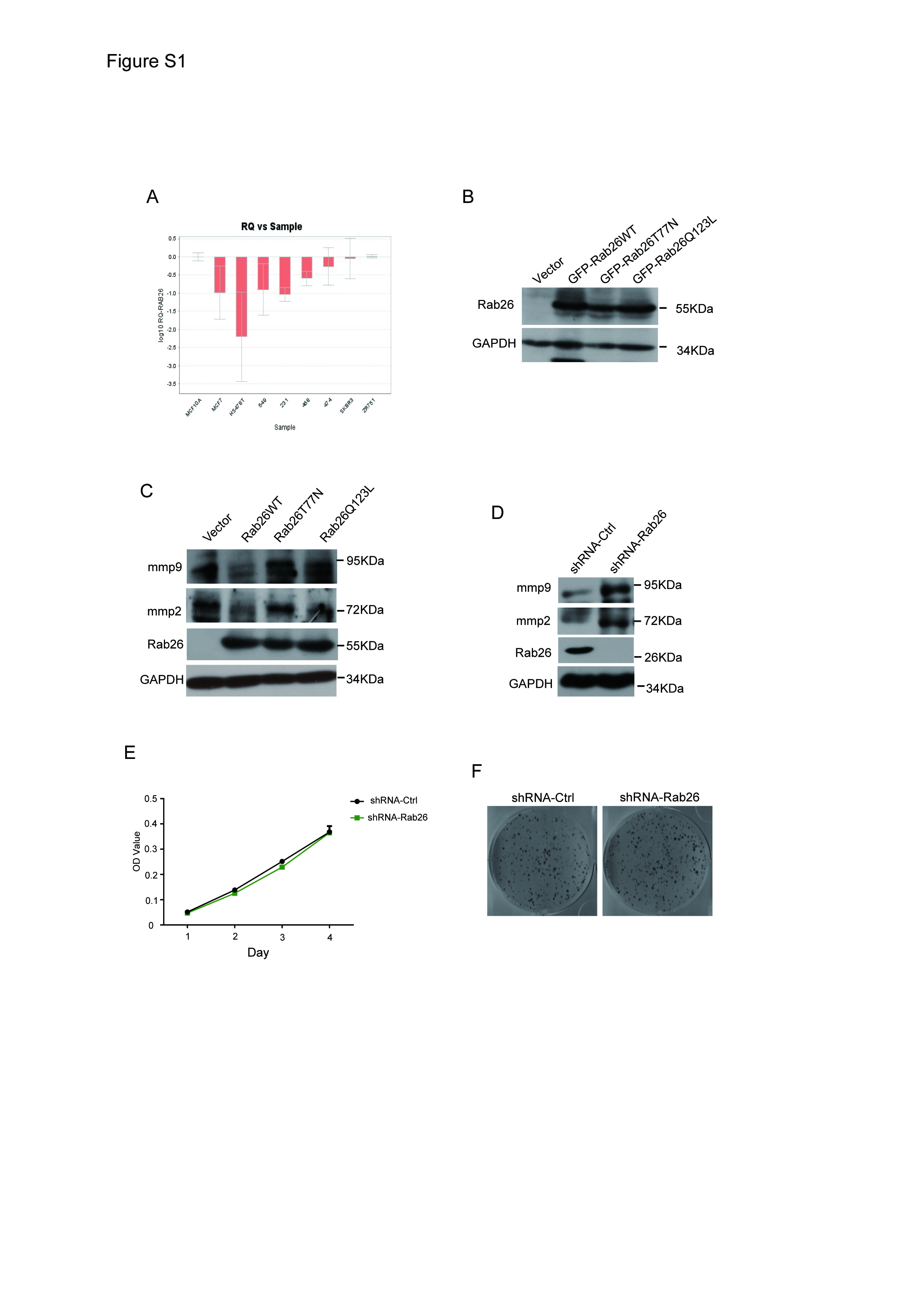

Supplement: Supplementary file 3 — Figure S1 [file 41419_2021_3561_MOESM3_ESM.tif]

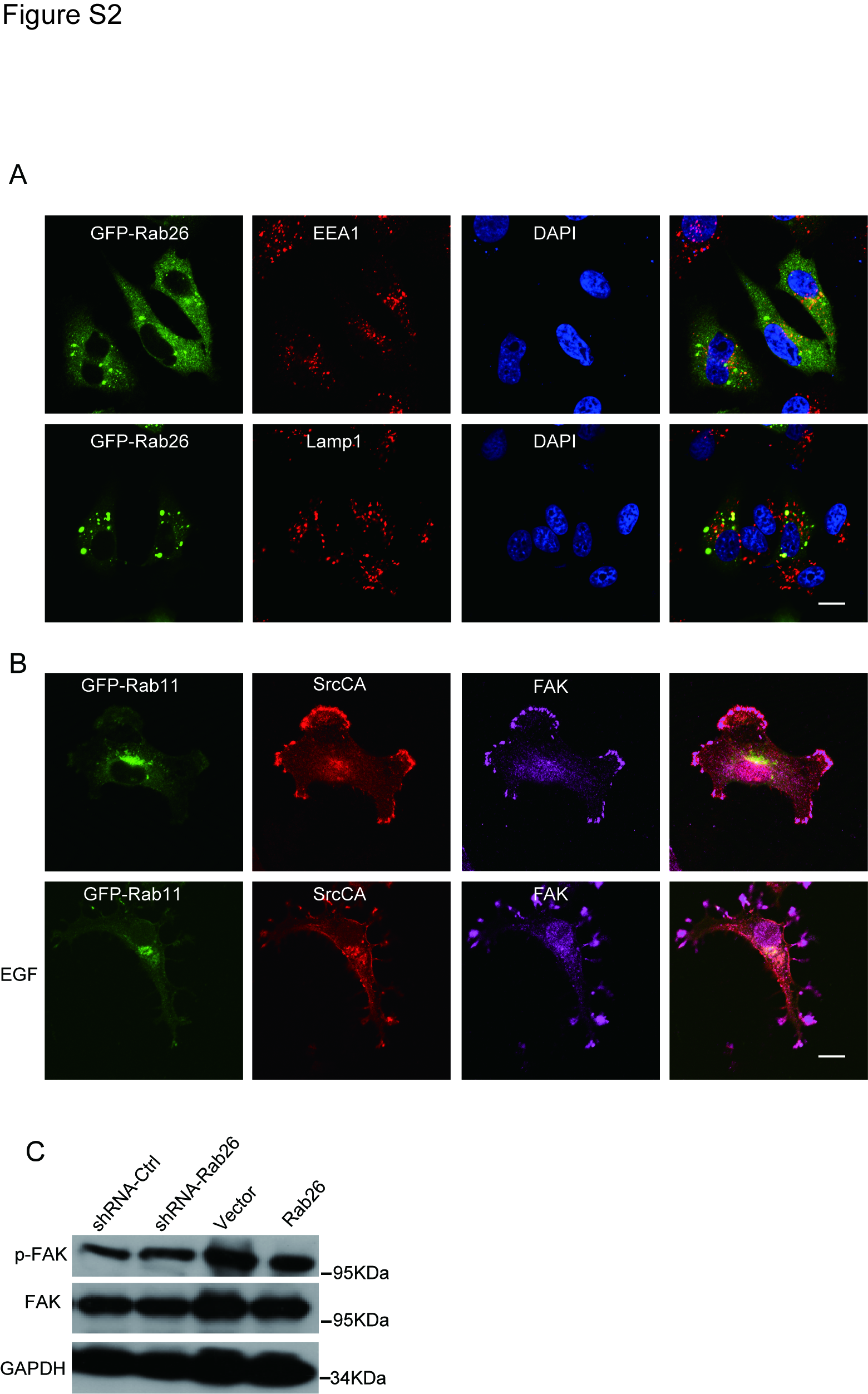

Supplement: Supplementary file 4 — Figure S2 [file 41419_2021_3561_MOESM4_ESM.tif]

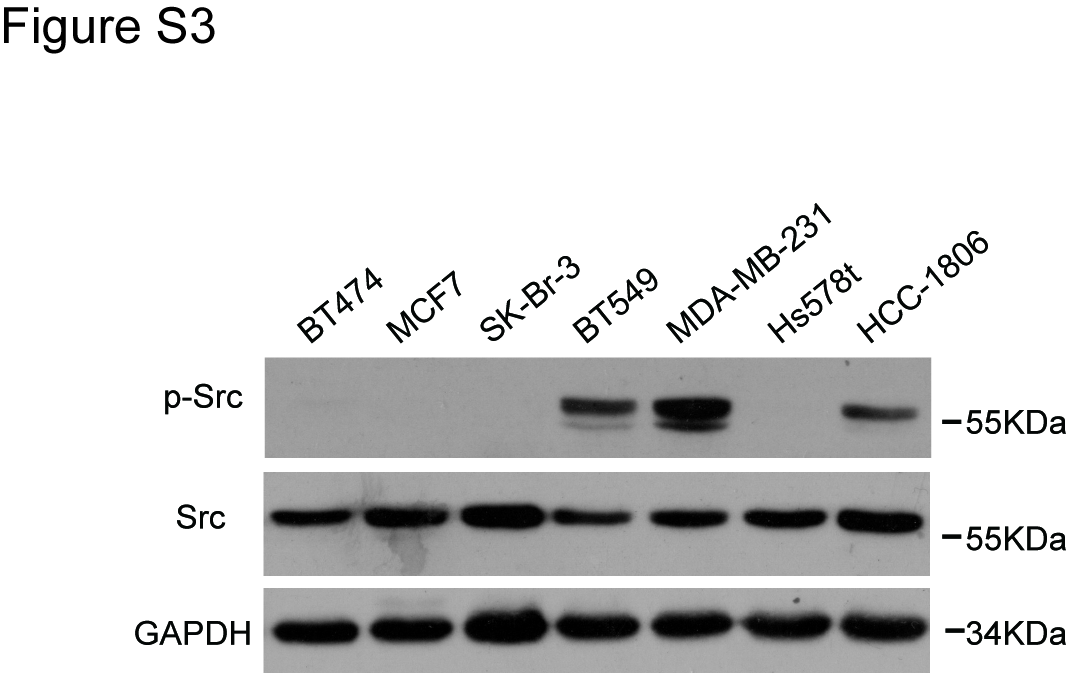

Supplement: Supplementary file 5 — Figure S3 [file 41419_2021_3561_MOESM5_ESM.tif]
