# Supplementary material for: Rab26 suppresses migration and invasion of breast cancer cells through mediating autophagic degradation of phosphorylated Src
Source: Cell Death Dis. 2021 Mar 17;12(4):284. doi: 10.1038/s41419-021-03561-7 (PMC7969620; doi:10.1038/s41419-021-03561-7)

Rab26 suppresses migration and invasion of breast cancer cells through mediating autophagic degradation of phosphorylated Src

Huiying Liu et al

The original Western blots Data

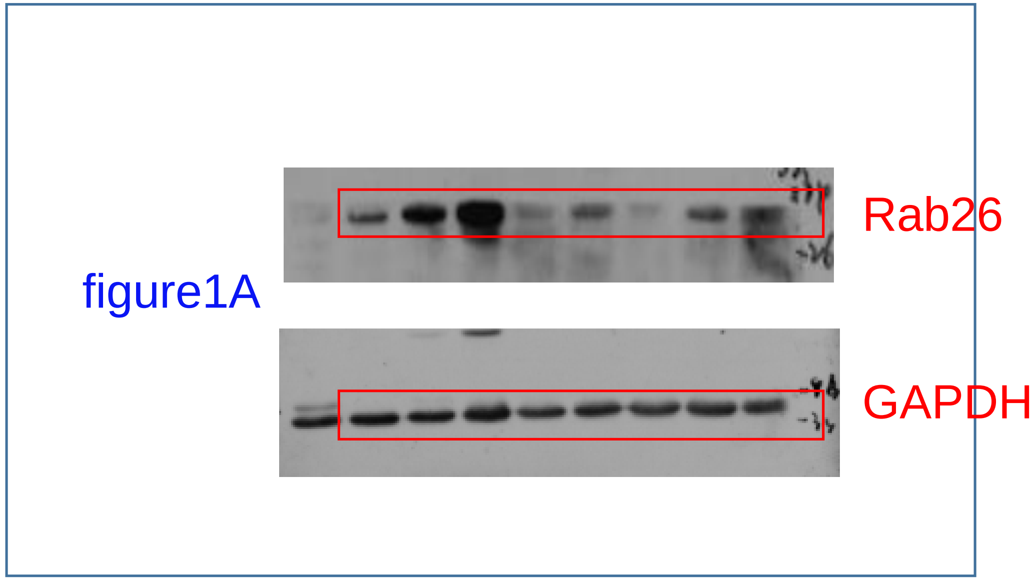

two figures share one internal reference

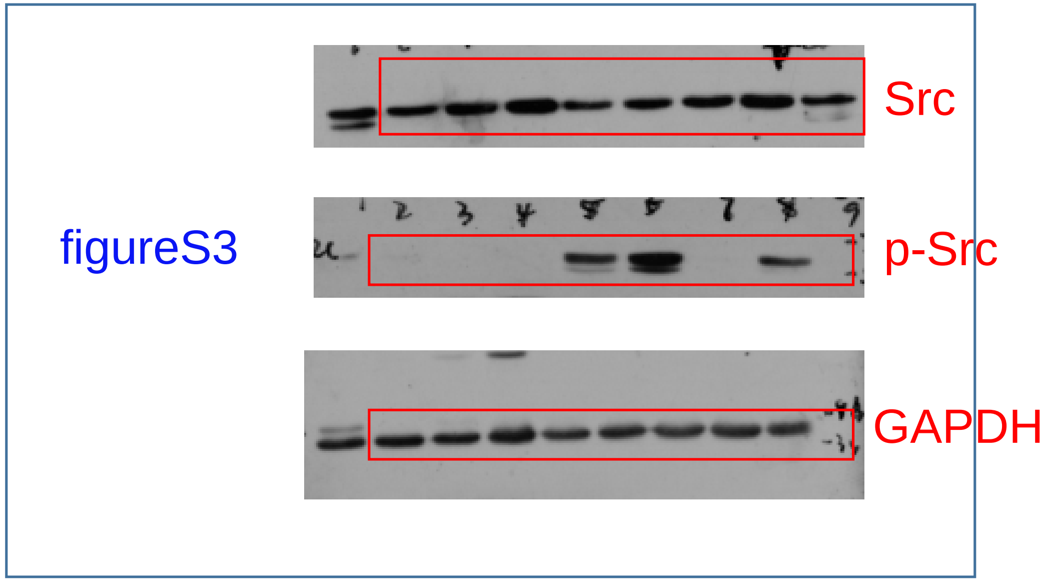

figureS1B

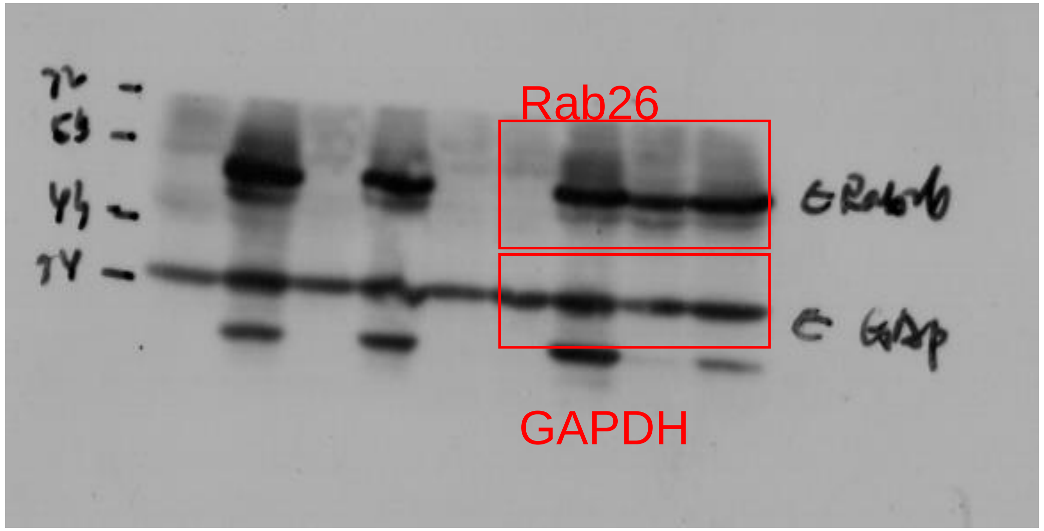

figure2A

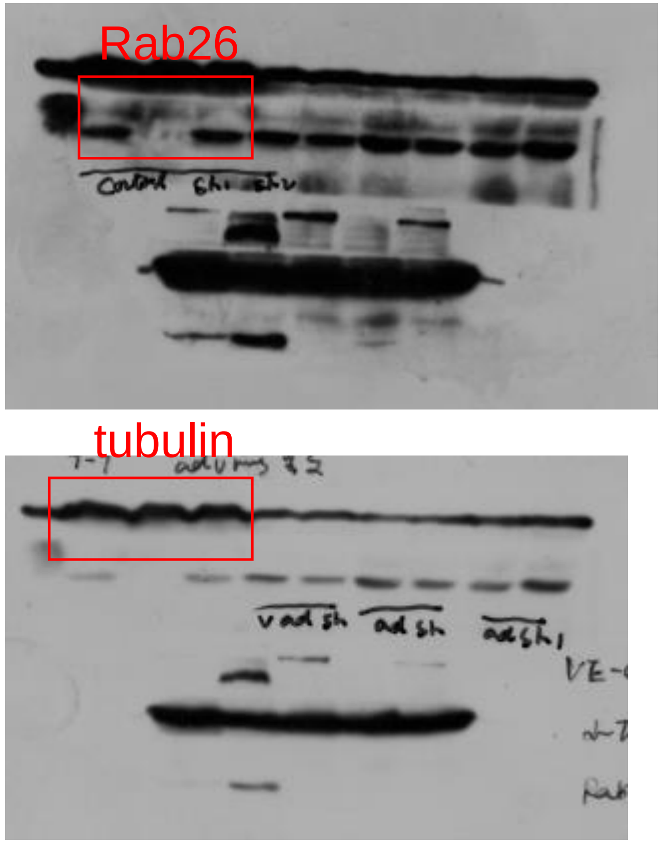

figure4B

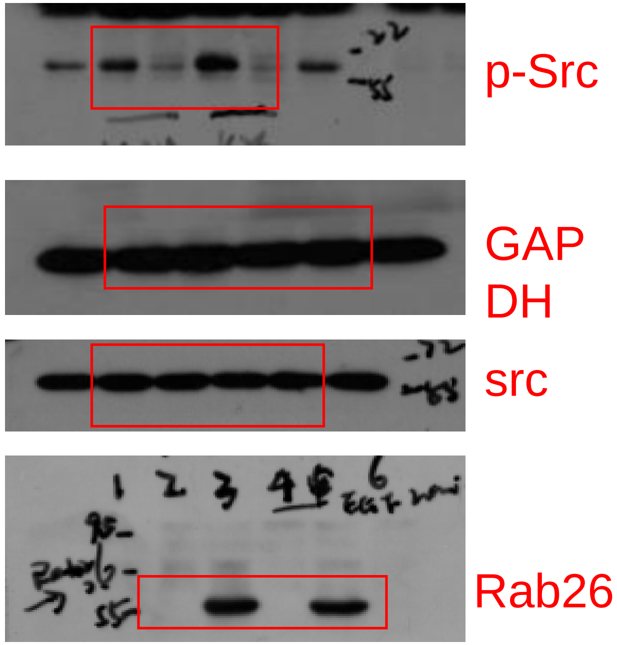

figure4D

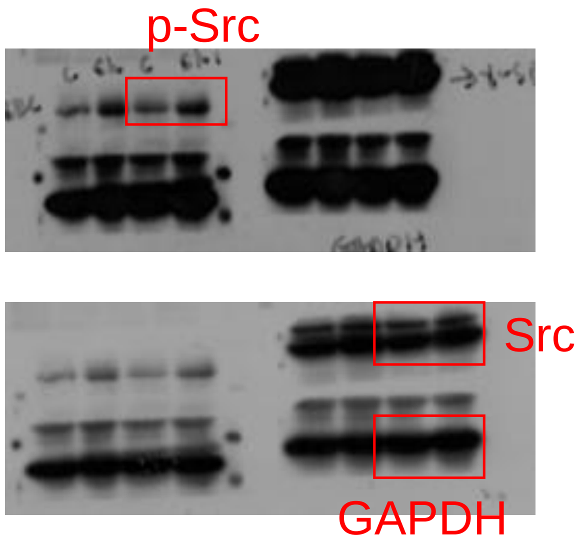

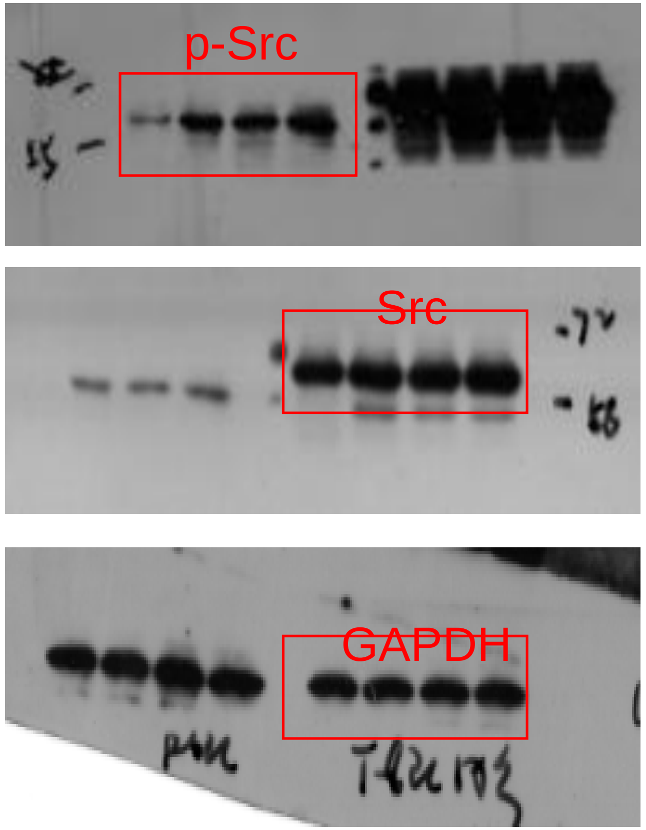

figure5A

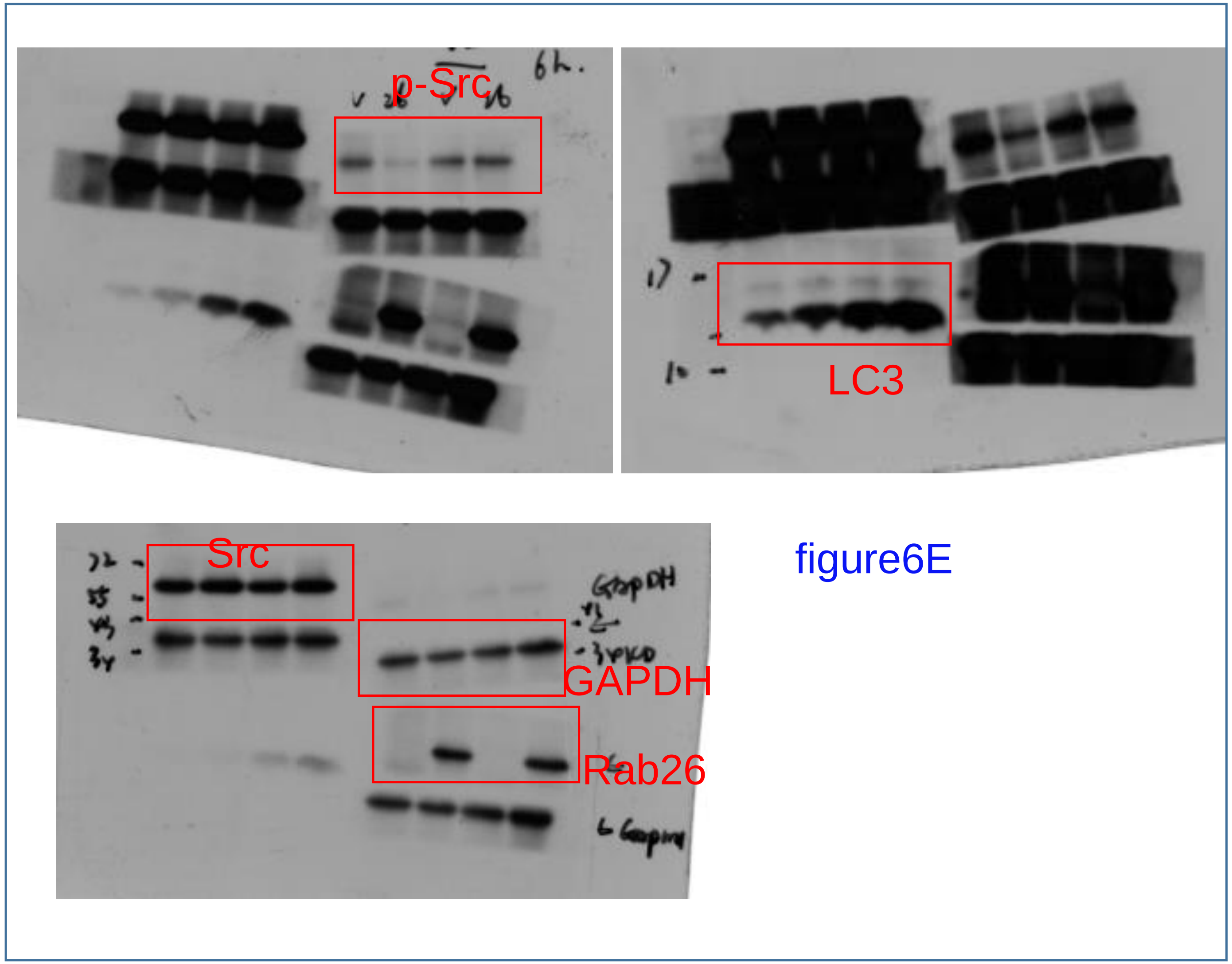
p-Src
LC3
Src
GAPDH
Rab26
figure6E

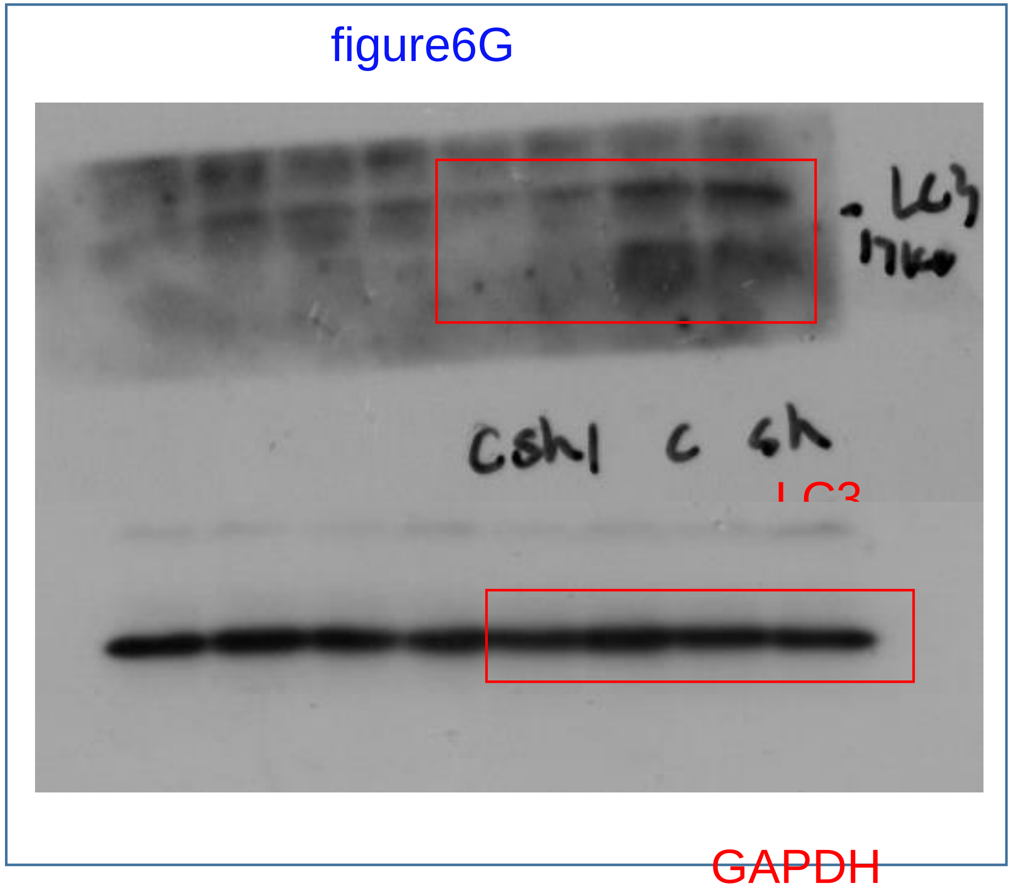
figure6G
LC3
17kD
GAPDH

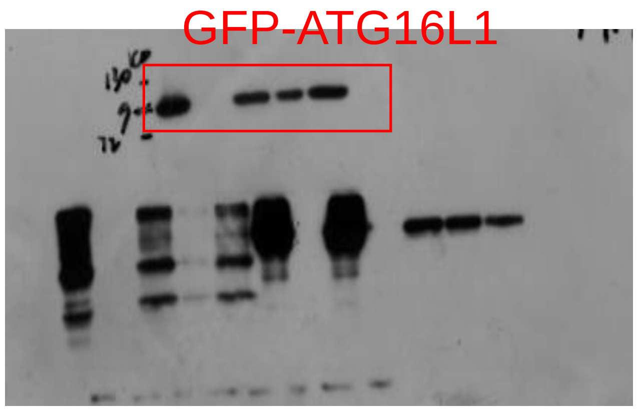

figure7A

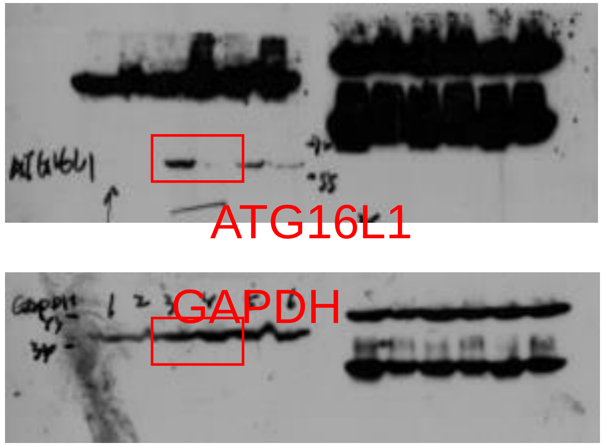

figure7B

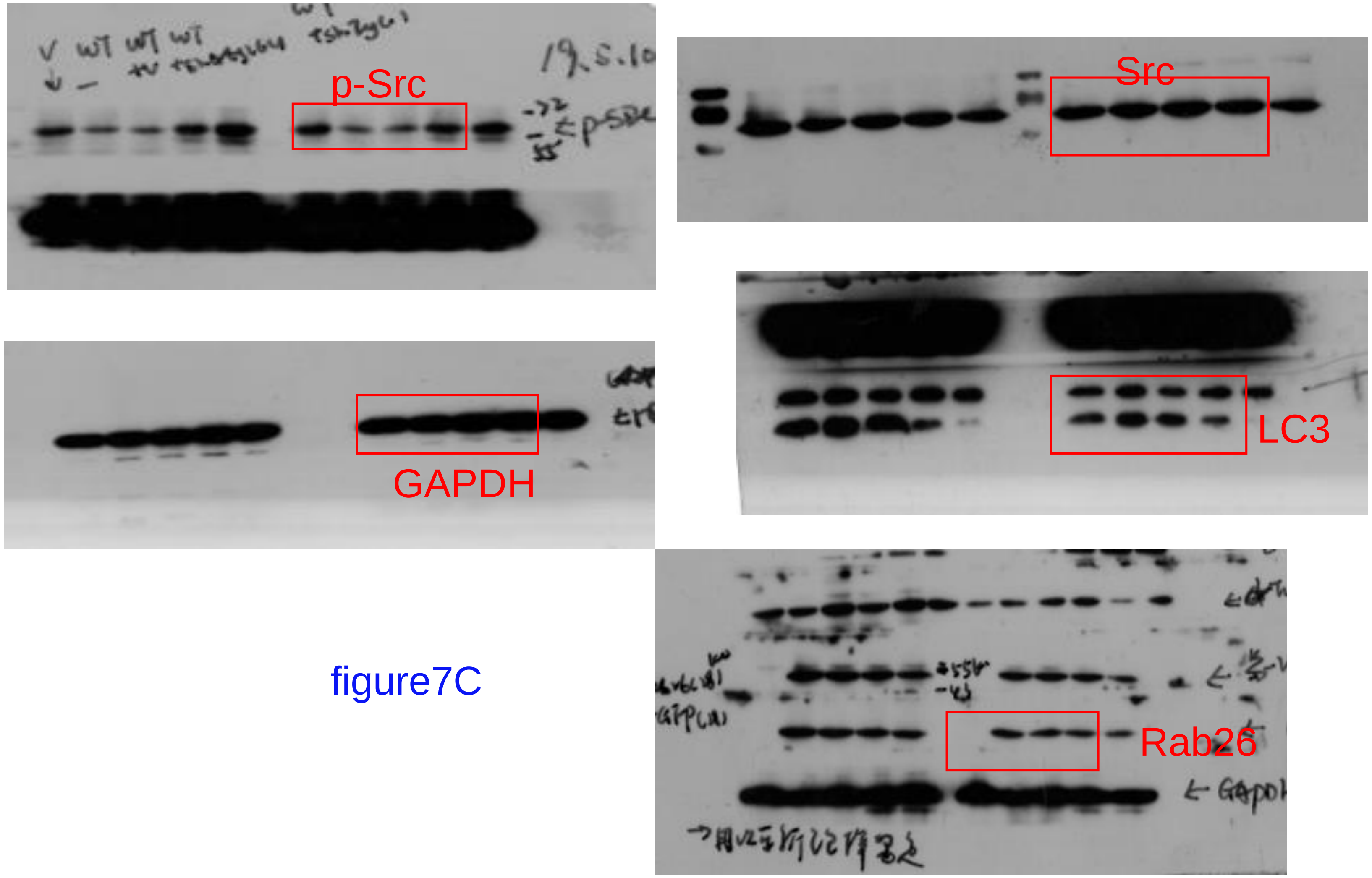

figure7C

figure8F

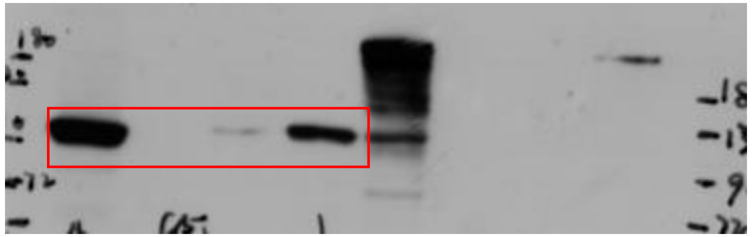

figure8.H

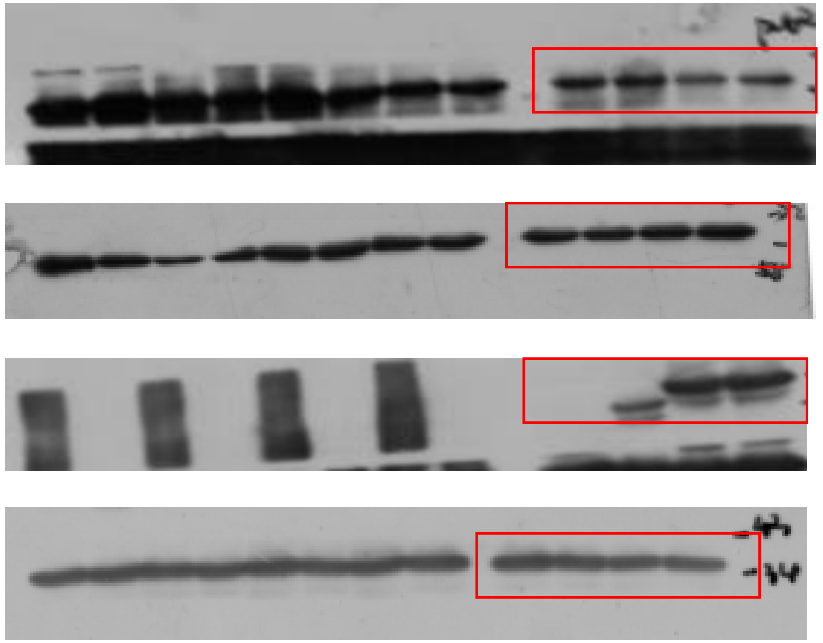

Supplement: Supplementary file 6 — Related Manuscript File [file 41419_2021_3561_MOESM6_ESM.pdf]
